# Genomic selection for genotype performance and stability using information on multiple traits and multiple environments

**DOI:** 10.1007/s00122-023-04305-1

**Published:** 2023-04-07

**Authors:** J. Bančič, B. Ovenden, G. Gorjanc, D. J. Tolhurst

**Affiliations:** 1 The Roslin Institute and Royal (Dick) School of Veterinary Studies, University of Edinburgh, Easter Bush, Midlothian UK; 2 NSW Department of Primary Industries, Wagga Wagga, NSW Australia

## Abstract

**Key message:**

The inclusion of multiple traits and multiple environments within a partially separable factor analytic approach for genomic selection provides breeders with an informative framework to utilise genotype by environment by trait interaction for efficient selection.

**Abstract:**

This paper develops a single-stage genomic selection (GS) approach which incorporates information on multiple traits and multiple environments within a partially separable factor analytic framework. The factor analytic linear mixed model is an effective method for analysing multi-environment trial (MET) datasets, but has not been extended to GS for multiple traits and multiple environments. The advantage of using all information is that breeders can utilise genotype by environment by trait interaction (GETI) to obtain more accurate predictions across correlated traits and environments. The partially separable factor analytic linear mixed model (SFA-LMM) developed in this paper is based on a three-way separable structure, which includes a factor analytic matrix between traits, a factor analytic matrix between environments and a genomic relationship matrix between genotypes. A diagonal matrix is then added to enable a different genotype by environment interaction (GEI) pattern for each trait and a different genotype by trait interaction (GTI) pattern for each environment. The results show that the SFA-LMM provides a better fit than separable approaches and a comparable fit to non-separable and partially separable approaches. The distinguishing feature of the SFA-LMM is that it will include fewer parameters than all other approaches as the number of genotypes, traits and environments increases. Lastly, a selection index is used to demonstrate simultaneous selection for overall performance and stability. This research represents an important continuation in the advancement of plant breeding analyses, particularly with the advent of high-throughput datasets involving a very large number of genotypes, traits and environments.

**Supplementary Information:**

The online version contains supplementary material available at 10.1007/s00122-023-04305-1.

## Introduction

This paper develops a single-stage genomic selection (GS) approach which incorporates information on multiple traits and multiple environments within a partially separable factor analytic framework. The factor analytic linear mixed model of Smith et al. (2001) is an effective method for analysing multi-environment trial (MET) datasets, which includes a parsimonious model for genotype by environment interaction (GEI). The factor analytic model has already been applied to multi-trait datasets in order to model genotype by trait interaction (GTI), but has not been extended to GS for multiple traits and multiple environments (Meyer 2007, 2009). The GS approach developed in this paper extends the factor analytic model to incorporate both sources of information.

Plant breeders select genotypes with superior performance across a set of production environments for multiple traits of commercial importance. Traditionally, threshold selection has been used by setting thresholds for each trait separately, but this ignores the genetic correlations between traits and may exclude genotypes that could serve as potential parents (e.g. high-yielding genotypes that are too tall for release). A more efficient approach is to use a selection index, which does consider the genetic correlations between traits and weights their importance based on the breeding objectives (Bernardo 2010). Selection indices have become a popular approach to advance material through the breeding pipeline for commercial release and to select potential parents for future crosses (Batista et al. 2021).

**Fig. 1:**
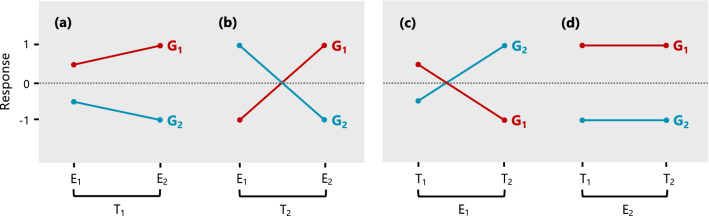
The response of hypothetical genotypes G1 and G2 measured in environments E1 and E2 for traits T1 and T2. The left panel demonstrates **a** non-crossover GEI for T1 and **b** crossover GEI for T2. The right panel demonstrates **c** a mixture of non-crossover and crossover GTI for E1 and **d** no GTI for E2. Collectively, all plots demonstrate GETI across all trait by environment combinations

Genomic selection is a form of marker-assisted selection that can improve the rate of genetic gain in plant breeding programs (Meuwissen et al. 2001). GS has already been used in the context of a selection index (Céron-Rojas and Crossa 2018), however, many of the current applications only use information on multiple environments for a single trait or multiple traits for a single environment, and this may limit the potential genetic gain. The advantage of using all information is that breeders can utilise genotype by environment by trait interaction (GETI), which reflects the differential response of genotypes to different trait by environment combinations (see Fig. 1). Another advantage is that breeders can obtain more accurate predictions across correlated traits and environments, regardless of whether phenotypic data are available on all genotype by environment by trait combinations. This is especially appealing for traits and environments with low heritability or traits which are difficult/expensive to phenotype.

**Fig. 2:**
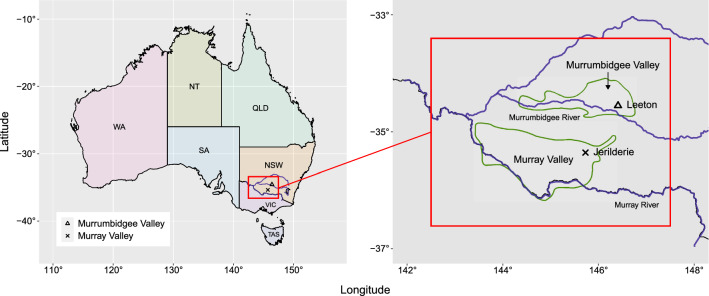
Map of the Australian rice growing regions in the multi-trait MET dataset, including the location of trials at Leeton in the Murrumbidgee Valley and Jerilderie in the Murray Valley

**Fig. 3:**
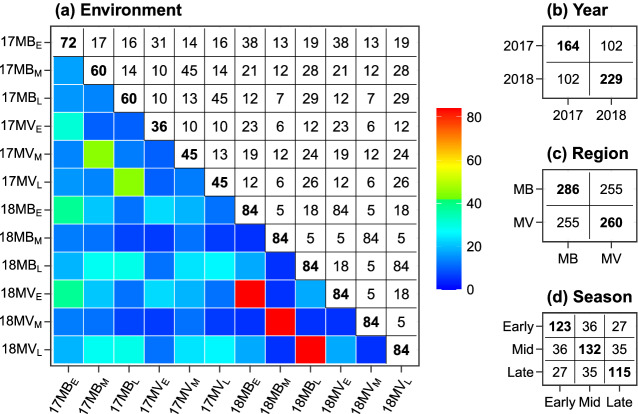
Connectivity in the multi-trait MET dataset in terms of the number of genotypes in common between pairs of **a** environments, **b** years, **c** regions and **d** seasons. The diagonal elements in all plots are the number of unique genotypes and the lower diagonal in **a** is a heatmap with supporting colorkey

Genomic selection for multiple traits and multiple environments was first considered by Montesinos-López et al. (2016). They demonstrated a *separable* model for GETI, which assumes complete separability between traits, environments and genotypes so it can be written as the Kronecker product of three variance matrices. Separable models are appealing because they have fewer variance parameters than equivalent non-separable models and they capture the factorial structure in the data. The separable model of Montesinos-López et al. (2016) includes the Kronecker product of an unstructured matrix between traits, a diagonal matrix between environments and a genomic relationship matrix between genotypes. Montesinos-López et al. (2019) extended this model to include an unstructured matrix between traits as well as between environments. They used biplots to explore GETI, which display the same GEI pattern for each trait and the same GTI pattern for each environment. This example highlights an important limitation of using separable models for multiple traits and multiple environments, that is they provide a very restrictive model for GETI.

An alternative approach is to use a *non-separable* model for GETI, which is fitted directly to the individual trait by environment combinations. Non-separable models are appealing because they provide a more general framework for GETI that enables different GEI and different GTI patterns. Smith et al. (2007) demonstrated a non-separable factor analytic linear mixed model which is an extension of the single-trait approach of Smith et al. (2001). The advantage of using factor analytic models is that they provide a parsimonious alternative to the unstructured model in terms of a small number of common factors. The advantage of using non-separable factor analytic models is that they can be applied to datasets with very few trait by environment combinations in order to exploit common information shared across either source. However, the non-separable factor analytic model has more variance parameters than an equivalent separable model and it does not provide direct predictions for those trait by environment combinations without phenotypic data.

Recently, Smith et al. (2019) demonstrated a *partially separable* model for GETI, which exploits the appealing features of the separable and non-separable models. Their model includes the Kronecker product of an unstructured matrix between traits and a factor analytic matrix between environments, with an additional diagonal matrix that captures variation specific to traits. This model enables different GEI and different GTI patterns while maintaining fewer variance parameters than an equivalent non-separable model. Smith et al. (2019) also demonstrated the application of plant breeding selection tools, where genotypes with high overall performance and stability are of high interest to breeders (also see Smith and Cullis 2018). There are two limitations of this approach: (i) the unstructured matrix becomes computationally prohibitive for a typical number of traits and (ii) only variation specific to traits is considered, but not environments.

The aim of this paper is to extend the partially separable approach of Smith et al. (2019) for GS using a factor analytic matrix between traits as well as between environments, with an additional diagonal matrix that captures variation specific to traits as well as environments. This new approach is hereafter referred to as the partially separable factor analytic linear mixed model (SFA-LMM). The utility of the SFA-LMM is compared to the non-separable approach of Smith et al. (2007), the partially separable approach of Smith et al. (2019) and the two separable approaches of Montesinos-López et al. (2016, 2019) using a multi-trait MET dataset from the Australian Rice Breeding Program. Lastly, a selection index is used to demonstrate simultaneous selection for overall performance and stability, which extends the plant breeding selection tools of Smith and Cullis (2018).

## Materials and methods

### Data description

The Australian Rice Breeding Program evaluates the commercial merit of test genotypes by annually conducting multi-environment field trials for multiple traits. Only a single late-stage of field evaluation is considered in this paper, with phenotypes collected on grain yield (YLD; t/ha), days to flowering (DTF) and plant height (PHT; cm). The multi-trait MET dataset for 2017-18 is summarised in Tables 1, 2 and Figs.  2, 3.

**Table 1:** Summary of growing environments in the multi-trait MET dataset

Region	Env^*^	Genotypes				YLD (t/ha)			DTF (days)			PHT (cm)		
		Total	2rep	>2rep	Plots	Mean	$h^2$	NAs	Mean	$h^2$	NAs	Mean	$h^2$	NAs
$\scriptstyle \triangle$ Murrumbidgee	17MB$_\text {E}$	72	24	48	240	8.6	0.54	2	98.3	0.57	8	80.5	0.79	1
	17MB$_\text {M}$	60	0	60	240	8.5	0.61	5	91.7	0.54	0	80.3	0.53	0
	17MB$_\text {L}$	60	0	60	240	6.7	0.19	4	83.7	0.46	5	82.3	0.46	0
	18MB$_\text {E}$	84	84	0	252	11.5	0.48	18	110.2	0.72	0	92.3	0.54	0
	18MB$_\text {M}$	84	84	0	252	8.8	0.75	5	95.9	0.19	0	84.8	0.50	1
	18MB$_\text {L}$	84	84	0	252	10.0	0.35	27	90.2	0.48	4	84.7	0.50	0
$\times$ Murray Valley	17MV$_\text {E}$	36	36	0	108	9.6	0.55	0	120.3	0.75	1	75.7	0.90	0
	17MV$_\text {M}$	45	45	0	135	10.3	0.44	1	117.9	0.46	0	68.7	0.60	1
	17MV$_\text {L}$	45	45	0	135	7.9	0.71	1	106.4	0.82	10	71.0	0.75	1
	18MV$_\text {E}$	84	84	0	168	9.6	0.41	10	124.0	0.55	17	75.6	0.40	0
	18MV$_\text {M}$	84	84	0	168	10.3	0.35	2	115.4	0.48	5	73.5	0.29	0
	18MV$_\text {L}$	84	84	0	168	6.7	0.36	5	104.3	0.40	20	63.7	0.35	1
**Overall**	–	**291**	–	–	**2358**	**9.0**	**0.45**	**80**	**104.9**	**0.54**	**70**	**77.7**	**0.55**	**5**

Presented for each environment is the number of genotypes (with two or more replicates) and number of plots. Also presented for grain yield, days to flowering and plant height is the mean, generalised narrow-sense heritability ($h^2$, Oakey et al. 2006) and number of missing values

^*^Each environment corresponds to a unique year-region-season combination

**Table 2:** Summary of agronomic traits in the multi-trait MET dataset

		YLD (t/ha)			DTF (days)			PHT (cm)		
		Min	Mean	Max	Min	Mean	Max	Min	Mean	Max
Year	2017	2.0	8.4	12.5	68.0	99.2	128.0	56.0	77.8	140.0
	2018	1.6	9.6	15.6	73.0	104.8	127.0	49.0	80.7	129.0
Region	$\scriptstyle \triangle$ Murrumbidgee	2.0	9.0	15.6	68.0	95.1	122.0	62.0	84.2	140.0
	$\times$ Murray Valley	1.6	9.0	12.5	86.0	114.7	128.0	49.0	71.2	99.0
Season	Early	2.0	10.5	15.6	99.0	116.4	128.0	65.0	83.6	129.0
	Mid	2.0	9.3	12.5	84.0	104.5	123.0	56.0	78.4	140.0
	Late	1.6	8.1	12.8	68.0	93.2	112.0	49.0	77.9	109.0

Presented for grain yield, days to flowering and plant height is the minimum, mean and maximum value for each year, region and season

*Note:* Values presented are prior to scaling phenotypes to unit variance

**Table 3:** Summary of the residual variance models considered in this paper

Model	Description	$\textbf{R}$		Parameters	Reference
*res*$_1$	Non-separable	$\oplus _{h=1}^{ps}\textbf{R}_h$	$\textbf{R}_h = \sigma ^2_{h}{\varvec{\Sigma }}_{\textbf{c}_h}\otimes {\varvec{\Sigma }}_{\textbf{r}_h}$	3*ps*	Smith et al. (2007)
*res*$_2$	Separable within envs	$\oplus _{j=1}^{p}\textbf{R}_j$	$\textbf{R}_j=\textbf{R}_{\textbf{t}_j}^\text {US}\otimes \textbf{R}_{\textbf{e}_j}$ $\textbf{R}_{\textbf{e}_j} = \sigma ^2_{j}{\varvec{\Sigma }}_{\textbf{c}_j}\otimes {\varvec{\Sigma }}_{\textbf{r}_j}$	$p[s(s+1)/2+2]$	Smith et al. (2007)
*res*$_3$	Separable across envs	$\mathbf {R_t^\text {US}}\otimes \mathbf {R_e}$	$\mathbf {R_e} = \oplus _{j=1}^{p}\textbf{R}_{\textbf{e}_j}$	$s(s+1)/2+3p-1$	
*res*$_4$	Partially separable	$\mathbf {R_t^\text {US}}\otimes \mathbf {R_e}+\mathbf {R_{te}}$	$\textbf{R}_{\textbf{te}}=\oplus _{h=1}^{ps}\sigma _{te_h}^2\textbf{I}_{n_h}$	$s(s+1)/2+p(s+3)-1$	This paper

Presented for each model is the form of the residual variance matrix ($\textbf{R}$), number of estimated variance parameters and the reference

*Note:* The spatial model ($\sigma ^2{\varvec{\Sigma }}_{\textbf{c}}\otimes {\varvec{\Sigma }}_{\textbf{r}}$) is chosen as a two-dimensional auto-regressive process of order one (Gilmour et al. 1997). Constraints are required in all separable and partially separable models to ensure identifiability during estimation. All models have standard ordering except for *res*$_2$ (traits within environments)

**Table 4:** Summary of the variance models for the additive GET effects considered in this paper

Type	Model	Description	$\mathbf {G_{te}}$	Parameters	References
Non-separable	*ndiag*	Non-separable diagonal	$\oplus _{h=1}^{ps}\sigma ^2_{h}$	*ps*	
	NFA*k*	Non-separable factor analytic	${{\varvec{\Lambda }}}\mathbf{D}{{\varvec{\Lambda }}}^{\scriptscriptstyle \top } + {\varvec{\Psi }}$	$ps(k + 1)- k(k-1)/2$	Smith et al. (2007)
Separable	*sdiag*	Separable diagonal	$\big [\oplus _{i=1}^s\sigma ^2_{t_i}\big ] \otimes \big [\oplus _{j=1}^p\sigma ^2_{e_j}\big ]$	$p + s - 1$	
	*mdiag*	Main effects plus diagonal	$\textbf{G}_{\textbf{t}}^{\text {US}}\otimes \big [\textbf{J}_p + \oplus _{j=1}^p\sigma ^2_{e_j}\big ]$	$s(s+1)/2 + p$	Montesinos-López et al. (2016)
	*mus*$^*$	Main effects plus unstructured	$\textbf{G}_{\textbf{t}}^{\text {US}}\otimes \big [\textbf{J}_p + \textbf{G}_{\textbf{e}}^{\text {US}}\big ]$	$[s(s+1) + p(p+1)]/2-1$	Montesinos-López et al. (2019)
Partially separable	*comp*	Compound symmetry	$\textbf{G}_{\textbf{t}_1}^{\text {US}} \otimes \textbf{J}_p + \textbf{G}_{\textbf{t}_2}^{\text {US}} \otimes \textbf{I}_p$	$s(s+1)$	Volpato et al. (2019)
	UFA$k_e$	Unstructured factor analytic	$\textbf{G}_{\textbf{t}}^{\text {US}} \otimes {\varvec{\Lambda }}_{\textbf{e}}\textbf{D}_{\textbf{e}}{\varvec{\Lambda }}_{\textbf{e}}^{\scriptscriptstyle \top } + {\varvec{\Psi }}_{\textbf{t}} \otimes \textbf{I}_p$	$s(s + 3)/2 + pk_e - k_e(k_e-1)/2 -1$	Smith et al. (2019)
	SFA$k_t$-$k_e$	Partially separable factor analytic	${\varvec{\Lambda }}_{\textbf{t}}\textbf{D}_{\textbf{t}}{{\varvec{\Lambda }}}^{\scriptscriptstyle \top }_{\textbf{t}} \otimes {{\varvec{\Lambda }}}_{\textbf{e}}\textbf{D}_{\textbf{e}}{{\varvec{\Lambda }}}^{\scriptscriptstyle \top }_{\textbf{e}} + {\varvec{\Psi }}_{\textbf{t}}\otimes {\varvec{\Psi }}_{\textbf{e}}$	$s(k_t + 1) - k_t(k_t-1)/2$	This paper
				$$+ p(k_e + 1) - k_e(k_e-1)/2 -2$$	

Presented for each model is the form of the additive genetic variance matrix between trait by environment combinations ($\mathbf {G_{te}}$), number of estimated variance parameters and the reference

*Note:* The *vps*-vector of additive GET effects is given by $\mathbf {u_a}$, with var$(\mathbf {u_a})=\mathbf {G_{te}}\otimes \mathbf {G_g}$, where $\textbf{G}^{ps\times ps}_{\textbf{te}}$ is either a non-separable, separable or partially separable matrix between trait by environment combinations and $\textbf{G}^{v\times v}_{\textbf{g}}$ is a genomic relationship matrix between genotypes. Constraints are required in all separable and partially separable models to ensure identifiability during estimation

$^*$Equivalent to a separable unstructured model

#### Experimental design and phenotypic data

A total of 291 genotypes were evaluated across 12 environments in the Murrumbidgee Valley and Murray Valley rice growing regions of Australia (Table 1, Fig. 2). Each environment comprised a single field trial, and is indexed by one of two years (2017 or 2018), one of two regions (Murrumbidgee or Murray Valley) and one of three seasons (early, mid or late). Each trial was designed as a randomised complete block design or incomplete block design (17MB$_\text {E}$ only) with 2–4 blocks of 36–84 genotypes for a total of 108–252 plots. Four check cultivars were evaluated in all 12 environments with phenotypes recorded on 36 plots each. The remaining 287 test genotypes were evaluated in 1–12 environments (mean of 3) with phenotypes recorded on 2–36 plots each (mean of 8). The number of genotypes in common between environments ranged from 5 to 84, with mean of 19 (Fig. 3). The number of genotypes in common between years (102), regions (255) and seasons (27–36) produced good connectivity across these factors. All traits were recorded in all environments for all genotypes grown, except for missing values (Table 2). There are considerable phenotypic differences between years, regions and seasons for all traits, but especially between regions for DTF and PHT and between seasons for YLD and DTF. The phenotypes for each trait were then scaled to unit variance for model fitting, with model parameters transformed back to their original scale after estimation.

**Table 5:** Non-separable linear mixed models fitted to the multi-trait MET dataset

(a) Baseline linear mixed models				(b) Non-separable factor analytic linear mixed models				
rModel	rPars	Loglik	AIC	aModel	aPars	Loglik	AIC	$\bar{v}$
*res*$_1$	108	3391.8	− 6315.7	NFA1	72	3686.3	− 6833.0	60.1
*res*$_2$	96	3344.6	− 6245.2	NFA2	107	3780.0	− 6940.0	76.3
*res*$_3$	41	3118.1	− 5902.2	**NFA3**	**141**	**3834.7**	**− 6981.5**	**90.0**
***res***$_{\mathbf{4}}$	**77**	**3418.4**	**− 6430.9**	NFA4	174	3875.3	− 6993.0	91.9

Presented for each model is the number of estimated residual or additive genetic variance parameters, residual log-likelihood, AIC and overall percentage of variance explained ($\bar{v}$)

*Note:* A non-separable diagonal model (*ndiag*) is used for the additive and non-additive GET effects in all baseline linear mixed models and a non-separable factor analytic model of order one (NFA1) is used for the non-additive GET effects in all factor analytic linear mixed models. The selected models are distinguished with *bold font*

**Table 6:** Separable and partially separable linear mixed models fitted to the multi-trait MET dataset

(a) Unstructured factor analytic linear mixed models					(b) Partially separable factor analytic linear mixed models				
aModel	aPars	Loglik	AIC	$\bar{v}$	aModel	aPars	Loglik	AIC	$\bar{v}$
*sdiag*	14	3355.0	− 6348.0	–	*comp*	12	3559.1	− 6760.2	74.6
*mdiag*	18	3571.0	− 6772.0	75.3	SFA1-1	28	3618.4	− 6774.7	62.7
*mus*	83	−	–	–	SFA2-1	30	3671.8	− 6877.6	65.8
UFA1	20	3672.8	− 6899.6	80.1	SFA3-1	31	3687.9	− 6907.8	77.0
**UFA2**	**31**	**3706.3**	**− 6944.5**	**83.2**	**SFA3-2**	**42**	**3726.4**	**− 6962.7**	**83.0**
UFA3	41	3715.0	− 6942.1	85.3	SFA3-3	52	3731.1	− 6952.3	84.3

Presented for each model is the number of estimated additive genetic variance parameters, residual log-likelihood, AIC and overall percentage of variance explained ($\bar{v}$)

*Note:* A non-separable factor analytic model of order one (NFA1) is used for the non-additive GET effects in all factor analytic linear mixed models, whereas a non-separable diagonal model (*ndiag*) is used in all remaining linear mixed models. The percentage of variance explained for *comp* and *mdiag* represents the variance explained by the main effects alone. The selected models are distinguished with *bold font*

**Table 7:** The SFA3-2 model, Part 1: Summary of agronomic traits and growing environments

(a) Traits	Var	${v}_{{t}_i}$	$\hat{\varvec{\lambda }}^*_{\textbf{t}_1}$	$\hat{\varvec{\lambda }}^*_{\textbf{t}_2}$	$\hat{\varvec{\lambda }}^*_{\textbf{t}_3}$	(b) Envs	Var	${v}_{{e}_j}$	$\hat{\varvec{\lambda }}^*_{\textbf{e}_1}$	$\hat{\varvec{\lambda }}^*_{\textbf{e}_2}$
YLD	0.45	59.0	1.00	0.00	0.00	17MB$_\text {E}$	13.04	100.0	0.59	0.00
DTF	5.32	100.0	− 0.16	5.13	0.00	17MB$_\text {M}$	5.30	81.2	0.35	0.11
PHT	18.35	90.2	− 2.78	− 3.07	− 8.14	17MB$_\text {L}$	2.74	100.0	0.26	0.08
**Overall**	**8.04**	**83.0**	–	–	–	17MV$_\text {E}$	5.89	85.2	0.39	− 0.01
						17MV$_\text {M}$	6.53	99.7	0.34	0.24
						17MV$_\text {L}$	6.23	59.8	0.27	0.21
						18MB$_\text {E}$	16.86	89.1	0.59	0.32
						18MB$_\text {M}$	13.52	100.0	0.60	− 0.09
						18MB$_\text {L}$	8.18	82.4	0.40	0.22
						18MV$_\text {E}$	3.70	68.4	0.23	0.17
						18MV$_\text {M}$	6.03	62.6	0.33	0.13
						18MV$_\text {L}$	8.44	68.2	0.35	0.26
						**Overall**	**8.04**	**83.0**	–	–

Presented for each trait and environment is the additive genetic variance, percentage of variance explained $({v}_{{t}_i} \text{ and } {v}_{{e}_j})$ and the REML estimates of constrained factor loadings $(\hat{\varvec{\lambda}}^*_{\textbf{t}_l}$ and $\hat{\varvec{\lambda}}^*_{\textbf{e}_l})$. The traits are grain yield, days to flowering and plant height

*Note:* The overall additive genetic variance and percentage of variance explained ($\bar{v}$) are presented in the final row

#### Marker data

Marker data were available for 267 (of the 291) genotypes. Markers were coded as −1, 0 or 1 according to a set of 14,800 single–nucleotide polymorphisms generated using DArTseq (Sansaloni et al. 2011). The frequency of heterozygous markers was low given the level of selfing accumulated up to the late stage of evaluation. Markers were filtered using the *pedicure* package in *R* (Butler 2019), with minor allele frequency > 3% and missing value frequency < 20%. A total of 3895 markers were retained using this criteria. Missing values were imputed using the *k*-nearest neighbour approach of Troyanskaya et al. (2001) with $k = 10$. The genomic relationship matrix was constructed following VanRaden (2008) method 1. The diagonal elements ranged from 0.667 to 4.081, with mean of 1.512. The off-diagonal elements ranged from − 0.905 to 2.927, with mean of − 0.006.

### Statistical models

Assume the multi-trait MET dataset comprises $s=3$ traits, $p = 12$ environments and $v = 267$ genotypes with $r = 3895$ markers and $n = 2358$ plots in total. Let the *ns*-vector of scaled phenotypic data be given by $\textbf{y}=(\textbf{y}_1^{\scriptscriptstyle \top }, \ldots , \textbf{y}_s^{\scriptscriptstyle \top })^{\scriptscriptstyle \top }$, where $\textbf{y}_{i}=(\textbf{y}_{i1}^{\scriptscriptstyle \top }, \ldots , \textbf{y}_{ip}^{\scriptscriptstyle \top })^{\scriptscriptstyle \top }$ is the *n*-vector for the $i^{th}$ trait and $\textbf{y}_{ij}$ is the $n_{j}$-vector for the $i^{th}$ trait and $j^{th}$ environment, which is hereafter referred to as the $h^{th}$ trait by environment combination. The length of $\textbf{y}$ is therefore given by $ns = \sum _{h=1}^{ps} n_{h}.$

The linear mixed model for $\textbf{y}$ can be written as:

1 $$\begin{aligned} \textbf{y} = \textbf{X}\varvec{\tau } + \big (\textbf{I}_s \otimes \textbf{Z}\big )\textbf{u} + \big (\textbf{I}_s \otimes \mathbf {Z_p}\big )\mathbf {u_p} +\textbf{e}, \end{aligned}$$

where $\varvec{\tau }$ is a vector of fixed effects with design matrix $\textbf{X}$; $\textbf{u}$ is a *vps*-vector of random *genotype by environment by trait* (GET) effects with $n \times vp$ design matrix $\textbf{Z}$, $\mathbf {u_p}$ is a vector of random non-genetic effects with design matrix $\mathbf {Z_p}$, $\textbf{e}$ is the *ns*-vector of residuals and $\otimes$ is the Kronecker product operator. The effects in $\varvec{\tau }$ include the mean parameter for each trait, environment and their interaction. These effects capture the factorial structure between traits and environments, and also ensure the model is translational invariant. The effects in $\mathbf {u_p}$ accommodate the plot structures of individual environments for each trait (Bailey 2008). Further effects in $\varvec{\tau }$ and $\mathbf {u_p}$ relate to the 24 genotypes without marker data (Tolhurst et al. 2019) and spatial modelling (Gilmour et al. 1997).

It is assumed that:

$$\begin{aligned} \left[ \begin{array}{c} \textbf{u} \\ \mathbf {u_p} \\ \textbf{e} \end{array}\right] \sim \text{ N }\left( \left[ \begin{array}{c} \textbf{0} \\ \textbf{0} \\ \textbf{0} \end{array}\right] , \left[ \begin{array}{ccc} \textbf{G} &{} \textbf{0} &{} \textbf{0} \\ \textbf{0} &{} \ \mathbf {G_p} &{} \textbf{0} \\ \textbf{0} &{} \textbf{0} &{} \ \textbf{R} \end{array}\right] \right) , \end{aligned}$$

where $\textbf{G}$ is developed below and $\mathbf {G_p}=\oplus _{h=1}^{ps}\textbf{G}_{\textbf{p}_h}$ is a diagonal matrix in which $\textbf{G}_{\textbf{p}_h}$ is the variance component model for the $h^{th}$ trait by environment combination. More parsimonious forms of $\mathbf {G_p}$ can be used, including those which assume separability between traits and environments. Lastly, the form of $\textbf{R}$ is given by:

2 $$\begin{aligned} \textbf{R} = \textbf{R}_{\textbf{t}} \otimes \textbf{R}_{\textbf{e}} + \mathbf {R_{te}}, \end{aligned}$$

where $\textbf{R}_{\textbf{t}}$ is a $s \times s$ unstructured matrix between traits, $\textbf{R}_{\textbf{e}}=\oplus _{j=1}^p\textbf{R}_{\textbf{e}_j}$ is a $n \times n$ block diagonal matrix in which $\textbf{R}_{\textbf{e}_j}$ is the two-dimensional spatial model for the $j^{th}$ environment and $\textbf{R}_{\textbf{te}}=\oplus _{h=1}^{ps}\sigma _{te_h}^2\textbf{I}_{n_h}$ is a $ns \times ns$ diagonal matrix in which $\sigma _{te_h}^2$ is the residual specific variance for the $h^{th}$ trait by environment combination. This model provides a general framework for the residuals which enables a different correlation between traits for each environment and a different correlation between columns and rows for each trait by environment combination. This model is compared to three other residual variance models in “Results” (also see Table 3).

**Table 8:** The SFA3-2 model, Part 2: Pairwise additive genetic correlations between grain yield, days to flowering and plant height for each environment

Env	YLD-DTF	YLD-PHT	DTF-PHT
17MB$_\text {E}$	− 0.03	− 0.30	− 0.33
17MB$_\text {M}$	− 0.02	− 0.21	− 0.32
17MB$_\text {L}$	− 0.03	− 0.30	− 0.33
17MV$_\text {E}$	− 0.02	− 0.23	− 0.32
17MV$_\text {M}$	− 0.03	− 0.30	− 0.33
17MV$_\text {L}$	− 0.01	− 0.09	− 0.27
18MB$_\text {E}$	− 0.03	− 0.25	− 0.32
18MB$_\text {M}$	− 0.03	− 0.30	− 0.33
18MB$_\text {L}$	− 0.02	− 0.22	− 0.32
18MV$_\text {E}$	− 0.02	− 0.13	− 0.29
18MV$_\text {M}$	− 0.01	− 0.10	− 0.28
18MV$_\text {L}$	− 0.02	− 0.13	− 0.29
**Overall**	**− 0.02**	**− 0.20**	**− 0.31**

### Model for the GET effects

Following Tolhurst et al. (2019), the GET effects are partitioned into *additive* and *non-additive* GET effects:

3 $$\begin{aligned} \textbf{u}= \mathbf {u_a} + \mathbf {u_n} \ \ \qquad \text {and} \ \ \qquad \textbf{G}= \mathbf {G_a} + \mathbf {G_n}. \end{aligned}$$

The *additive* genetic variance matrix is given by $\mathbf {G_a}=\mathbf {G_{te}}\otimes \mathbf {G_g}$, where $\mathbf {G_{te}}$ is a $ps\times ps$ matrix between trait by environment combinations and $\mathbf {G_g}$ is a $v\times v$ genomic relationship matrix between genotypes (VanRaden 2008). The *non-additive* genetic variance matrix is given by $\mathbf {G_n}$, which is chosen as either a non-separable diagonal model or a non-separable factor analytic model of order one. Other forms of $\mathbf {G_n}$ can be used, including those which match $\mathbf {G_a}$. The forms of $\mathbf {G_a}$ considered below include:

- A non-separable model
- Two separable models
- Two partially separable models.

All variance models are summarised in Table 4, with full details provided below.

### Non-separable model

The *non-separable* model is fitted directly to the individual trait by environment combinations in $\mathbf {G_{te}}$. The variance matrix for $\mathbf {u_a}$ is therefore given by:

4 $$\begin{aligned} \mathbf {G_a} = \mathbf {G_{te}} \otimes \mathbf {G_g}, \end{aligned}$$

where $\mathbf {G_{te}}$ is a $ps \times ps$ non-separable matrix (Appendix 1). The non-separable model provides a general framework for GETI which enables a different GEI pattern for each trait and a different GTI pattern for each environment.

#### Non-separable factor analytic model

Smith et al. (2007) proposed a non-separable factor analytic model which is fitted directly to $\mathbf {G_{te}}$. This model is hereafter referred to as the NFA*k* model, where *k* denotes the number of common factors. The NFA*k* model is given by:

5 $$\begin{aligned} \mathbf {u_a}&= \big (\varvec{\lambda }_{1} \otimes \textbf{I}_v \big ) \textbf{f}_{1} + \ldots + \big (\varvec{\lambda }_{k} \otimes \textbf{I}_v \big ) \textbf{f}_{k} + \varvec{\delta } \nonumber \\&=\big ({\varvec{\Lambda }} \otimes \textbf{I}_v \big ) \textbf{f} + \varvec{\delta }, \end{aligned}$$

where ${\varvec{\Lambda }} = \big [\varvec{\lambda }_{1} \ldots \, \varvec{\lambda }_{k}\big ]$ is a $ps\times k$ non-separable matrix of trait by environment loadings, $\textbf{f} = \big ({\textbf{f}}_1^{{\scriptscriptstyle \top }}, \ldots , {\textbf{f}}_{k}^{{\scriptscriptstyle \top }}{\big )}^{\scriptscriptstyle \top }$ is a *vk*-vector of genotype scores and $\varvec{\delta } = \big ({\varvec{\delta }}_{1}^{{\scriptscriptstyle \top }},\, \ldots , {\varvec{\delta }}_{ps}^{{\scriptscriptstyle \top }}{\big )}^{\scriptscriptstyle \top }$ is a *vps*-vector of regression residuals which are specific to individual trait by environment combinations. This model is analogous to a multiple regression with *k* terms, where the loadings are latent covariates and the scores are regression slopes.

Following Smith et al. (2021), the loadings are assumed to have orthonormal columns, such that ${{\varvec{\Lambda }}}^{{\scriptscriptstyle \top }} {\varvec{\Lambda }}=\textbf{I}_k$. It is also assumed that:

$$\begin{aligned} \left[ \begin{array}{c} \textbf{f} \\ \varvec{\delta } \end{array}\right] \sim \text{ N }\left( \left[ \begin{array}{c} \textbf{0} \\ \textbf{0} \end{array}\right] , \left[ \begin{array}{cc} \textbf{D} &{} \, \textbf{0} \\ \textbf{0} &{} \, {\varvec{\Psi }} \end{array}\right] \otimes \mathbf {G_g}\right) , \end{aligned}$$

where $\textbf{D} = \oplus _{l=1}^{k}d_{l}$ is a diagonal matrix with score variances ordered as $d_1>\ldots > d_k$ and ${\varvec{\Psi }} = \oplus _{h=1}^{ps}\psi _{h}$ is a diagonal matrix with specific variances denoted by $\psi _{h}$. The variance matrix for $\mathbf {u_a}$ is therefore given by:

6 $$\begin{aligned} \mathbf {G_a}&= \big ({\varvec{\Lambda }} \mathbf{D}{\varvec{\Lambda }}^{\scriptscriptstyle \top } + {\varvec{\Psi }}\big ) \otimes \mathbf {G_g}. \end{aligned}$$

This variance matrix is an extension of Smith et al. (2007) for GS.

### Separable models

The *separable* models assume complete separability between traits and environments, such that $\mathbf {G_{te}}$ can be represented by the Kronecker product $\mathbf {G_t} \otimes \mathbf {G_e}$. The variance matrix for $\mathbf {u_a}$ can therefore be rewritten as:

7 $$\begin{aligned} \mathbf {G_a} = \mathbf {G_t} \otimes \mathbf {G_e} \otimes \mathbf {G_g}, \end{aligned}$$

where $\mathbf {G_t}$ is a $s\times s$ matrix and $\mathbf {G_e}$ is a $p\times p$ matrix (Appendix 1). Separable models have fewer variance parameters than non-separable models and capture the factorial structure between traits and environments. Note, however, separable models are restrictive since they assume the same GEI pattern for each trait and the same GTI pattern for each environment. The two separable models of Montesinos-López et al. (2016) and Montesinos-López et al. (2019) are considered in this paper (see Table 4).

### Partially separable models

The *partially separable* models maintain the appealing features of the separable models but fit a more general framework for GETI like the non-separable model. The two partially separable models below are obtained by fitting an additional variance for individual traits or individual traits and environments. This relaxes the assumption of separability and produces a structure that can no longer be represented by the Kronecker product of two matrices. The variance matrix for **u**_a_ can therefore be rewritten as:

8 $$\begin{aligned} \mathbf {G_a} = \mathbf {G_t} \otimes \mathbf {G_e} \otimes \mathbf {G_g} + \mathbf {D_{te}} \otimes \mathbf {G_g}, \end{aligned}$$

where **D**_**te**_ is a $ps \times ps$ diagonal matrix (Appendix 1).

#### Unstructured factor analytic model

Smith et al. (2019) proposed a partially separable model which includes an unstructured matrix for $\mathbf {G_t}$ and a factor analytic matrix for $\mathbf {G_e}$. This model is hereafter referred to as the UFA$k_e$ model, where $k_e$ denotes the number of common environmental factors. The UFA$k_e$ model is given by:

9 $$\begin{aligned} \mathbf {u_a}&= \big (\textbf{I}_s\otimes \varvec{\lambda }_{\textbf{e}_1}\otimes \textbf{I}_v\big )\textbf{f}_{\textbf{e}_1} +\ldots + \big (\textbf{I}_s\otimes \varvec{\lambda }_{\textbf{e}_{k_e}}\otimes \textbf{I}_v\big )\textbf{f}_{\textbf{e}_{k_e}} + \varvec{\delta }_{\textbf{t}} \nonumber \\&= \big (\textbf{I}_s\otimes {\varvec{\Lambda }}_{\textbf{e}}\otimes \textbf{I}_v\big )\mathbf {f_e} + \varvec{\delta }_{\textbf{t}}, \end{aligned}$$

where ${\varvec{\Lambda }}_{\textbf{e}} = \big [\varvec{\lambda }_{\textbf{e}_1} \ldots \, \varvec{\lambda }_{\textbf{e}_{k_e}}\big ]$ is a $p\times k_e$ matrix of environmental loadings, $\mathbf {f_{e}} = \big ({\textbf{f}}_{\textbf{e}_1}^{{\scriptscriptstyle \top }}, \ldots , {\textbf{f}}_{\textbf{e}_{k_e}}^{{\scriptscriptstyle \top }}{\big )}^{\scriptscriptstyle \top }$ is a $vk_e$-vector of genotype scores and $\varvec{\delta }_{\textbf{t}} = \big ({\varvec{\delta }}_{\textbf{t}_{1}}^{{\scriptscriptstyle \top }}, \ldots , {\varvec{\delta }}_{\textbf{t}_{s}}^{{\scriptscriptstyle \top }}{\big )}^{\scriptscriptstyle \top }$ is a *vps*-vector of regression residuals which are specific to individual traits. This model is analogous to a multiple regression with $k_e$ terms, where the loadings are latent covariates and the scores are regression slopes which are correlated across traits.

It is assumed that ${\varvec{\Lambda }}_{\textbf{e}}^{{\scriptscriptstyle \top }} {\varvec{\Lambda }}_{\textbf{e}}=\textbf{I}_{k_e}$, and that:

$$\begin{aligned} \left[ \begin{array}{c} \mathbf {f_{e}} \\ \varvec{\delta }_{\textbf{t}} \end{array}\right] \sim \text{ N }\left( \left[ \begin{array}{c} \textbf{0} \\ \textbf{0} \end{array}\right] , \left[ \begin{array}{cc} \mathbf {G_t} \otimes \mathbf {D_e} &{} \ \textbf{0} \\ \textbf{0} &{} \ {\varvec{\Psi }}_{\textbf{t}} \otimes \textbf{I}_p \end{array}\right] \otimes \mathbf {G_g}\right) , \end{aligned}$$

where $\mathbf {G_t}$ is a $s \times s$ unstructured matrix, $\mathbf {D_e} = \oplus _{l=1}^{k_e} d_{e_{l}}$ is a diagonal matrix with score variances ordered as $d_{e_1}>\ldots > d_{e_{k_e}}$ and ${\varvec{\Psi }}_{\textbf{t}} = \oplus _{i=1}^s\psi _{t_{i}}$ is a diagonal matrix with specific variances denoted by $\psi _{t_{i}}$. The variance matrix for $\mathbf {u_a}$ is therefore given by:

10 $$\begin{aligned} \mathbf {G_a}&= \big (\mathbf {G_t} \otimes {\varvec{\Lambda }}_{\textbf{e}} \mathbf {D_{e}}{\varvec{\Lambda }}_{\textbf{e}}^{\scriptscriptstyle \top } + {\varvec{\Psi }}_{\textbf{t}} \otimes \textbf{I}_p\big ) \otimes \mathbf {G_g}. \end{aligned}$$

This variance matrix is an extension of Smith et al. (2019) for GS.

#### Partially separable factor analytic model

The partially separable model developed below extends the UFA$k_e$ model to include a factor analytic matrix for $\mathbf {G_t}$ as well as a factor analytic matrix for $\mathbf {G_e}$. The variance matrix for $\mathbf {u_a}$ is therefore given by:

11 $$\begin{aligned} \mathbf {G_a} = \big ({\varvec{\Lambda }}_{\textbf{t}} \mathbf {D_t}{\varvec{\Lambda }}^{\scriptscriptstyle \top }_{\textbf{t}} + {\varvec{\Psi }}_{\textbf{t}}\big ) \otimes \big ({\varvec{\Lambda }}_{\textbf{e}} \mathbf {D_e}{\varvec{\Lambda }}^{\scriptscriptstyle \top }_{\textbf{e}} + {\varvec{\Psi }}_{\textbf{e}}\big ) \otimes \mathbf {G_g}, \end{aligned}$$

where ${\varvec{\Lambda }}_{\textbf{t}}= \big [\varvec{\lambda }_{\textbf{t}_1} \, \ldots \, \varvec{\lambda }_{\textbf{t}_{k_t}}\big ]$ is a $s\times k_t$ matrix of trait loadings and ${\varvec{\Lambda }}_{\textbf{e}}= \big [\varvec{\lambda }_{\textbf{e}_1} \, \ldots \, \varvec{\lambda }_{\textbf{e}_{k_e}}\big ]$ is a $p\times k_e$ matrix of environmental loadings, in which $k_t$ and $k_e$ denote the number of common trait and environmental factors, respectively. The loadings matrices are assumed to have orthonormal columns, such that ${\varvec{\Lambda }}_{\textbf{t}}^{\scriptscriptstyle \top }{\varvec{\Lambda }}_{\textbf{t}}=\textbf{I}_{k_t}$ and ${\varvec{\Lambda }}^{\scriptscriptstyle \top }_{\textbf{e}}{\varvec{\Lambda }}_{\textbf{e}}=\textbf{I}_{k_e}$. The diagonal matrices $\mathbf {D_{t}} = \oplus _{l=1}^{k_t} d_{t_l}$ and $\mathbf {D_{e}} = \oplus _{l=1}^{k_e} d_{e_l}$ include score variances for individual trait and environmental factors, while ${\varvec{\Psi }}_{\textbf{t}} = \oplus _{i=1}^s \psi _{t_i}$ and ${\varvec{\Psi }}_{\textbf{e}} = \oplus _{j=1}^p \psi _{e_j}$ include specific variances for individual traits and environments.

The separable variance matrix in Eq.  11 is restrictive because it assumes the same GEI pattern for each trait and the same GTI pattern for each environment. This matrix can be modified to enable different GEI and different GTI patterns:

12 $$\begin{aligned} \mathbf {G_a} = \big ({\varvec{\Lambda }}_{\textbf{t}}\mathbf {D_t}{\varvec{\Lambda }}^{\scriptscriptstyle \top }_{\textbf{t}} \otimes {\varvec{\Lambda }}_{\textbf{e}}\mathbf {D_e}{\varvec{\Lambda }}^{\scriptscriptstyle \top }_{\textbf{e}} + {\varvec{\Psi }}_{\textbf{t}}\otimes {\varvec{\Psi }}_{\textbf{e}}\big ) \otimes \mathbf {G_g}. \end{aligned}$$

This model is hereafter referred to as the SFA$k_t$-$k_e$ model. The SFA$k_t$-$k_e$ model for $\mathbf {u_a}$ is given by:

13 $$\begin{aligned} \mathbf {u_a} =& \ \big (\varvec{\lambda }_{\textbf{t}_1} \otimes \varvec{\lambda }_{\textbf{e}_1} \otimes \textbf{I}_v\big )\textbf{f}_{\textbf{te}_1} +\ldots \nonumber \\&\ +\big (\varvec{\lambda }_{\textbf{t}_{k_t}} \otimes \varvec{\lambda }_{\textbf{e}_{k_e}} \otimes \textbf{I}_v\big )\textbf{f}_{\textbf{te}_{k_tk_e}} + \varvec{\delta }_{\textbf{te}} \nonumber \\ =& \ \big ({\varvec{\Lambda }}_{\textbf{t}} \otimes {\varvec{\Lambda }}_{\textbf{e}} \otimes \textbf{I}_v\big )\mathbf {f_{te}} + \varvec{\delta }_{\textbf{te}} \end{aligned}$$

where ${\varvec{\Lambda }}_{\textbf{t}} \otimes {\varvec{\Lambda }}_{\textbf{e}}$ is the $ps \times k_tk_e$ separable matrix of trait and environmental loadings, $\mathbf {f_{te}}=\big ({\textbf{f}}_{\textbf{te}_{1}}^{{\scriptscriptstyle \top }}, \ldots , {\textbf{f}}_{\textbf{te}_{k_tk_e}}^{{\scriptscriptstyle \top }}{\big )}^{\scriptscriptstyle \top }$ is a $vk_tk_e$-vector of genotype scores and $\varvec{\delta }_{\textbf{te}} = \big ({\varvec{\delta }}_{\textbf{te}_1}^{{\scriptscriptstyle \top }},\, \ldots , {\varvec{\delta }}_{\textbf{te}_{ps}}^{{\scriptscriptstyle \top }}{\big )}^{\scriptscriptstyle \top }$ is a *vps*-vector of regression residuals which are specific to individual trait by environment combinations. This model is analogous to a multiple regression across two dimensions with $k_t$ and $k_e$ terms, where the loadings are latent covariates for each dimension and the scores are joint regression slopes across both dimensions.

Lastly, it follows from Eq.  12 that:

$$\begin{aligned} \left[ \begin{array}{c} \mathbf {f_{te}} \\ \varvec{\delta }_{\textbf{te}}\\ \end{array}\right] \sim \text{ N }\left( \left[ \begin{array}{c} \textbf{0} \\ \textbf{0} \end{array}\right] , \left[ \begin{array}{cc} \mathbf {D_t}\otimes \mathbf {D_e} &{} \textbf{0} \\ \textbf{0} &{} {\varvec{\Psi }}_{\textbf{t}} \otimes {\varvec{\Psi }}_{\textbf{e}} \end{array}\right] \otimes \mathbf {G_g}\right) .\end{aligned}$$

Other (non-separable) forms of the specific variance matrix can be used, including ${\varvec{\Psi }}_{\textbf{t}}\otimes\mathbf{I}_p + \mathbf{I}_s\otimes{\varvec{\Psi }}_{\textbf{e}}$ and ${\varvec{\Psi }} = \oplus _{h=1}^{ps}\psi _{h}$, but note that the former is not scale invariant and the latter may not be necessary for higher order models in which the variance explained by the regression residuals is small.

### Model estimation

All models for the additive and non-additive GET effects were implemented within the linear mixed model in Eq.  1 and fitted using *ASReml-R* (Butler 2022). The partially separable factor analytic linear mixed model (SFA-LMM) is given by:

14 $$\begin{aligned} \textbf{y} =& \ \textbf{X}\varvec{\tau } + \textbf{Z}_{{\varvec{\Lambda }}_{\textbf{te}}}\mathbf {f_{te}} + \big (\textbf{I}_s \otimes \textbf{Z}\big )\varvec{\delta }_{\textbf{te}} + \big (\textbf{I}_s \otimes \textbf{Z}\big )\mathbf {u_n} \, \nonumber \\&+\big (\textbf{I}_s \otimes \mathbf {Z_p}\big )\mathbf {u_p} + \textbf{e}, \end{aligned}$$

where $\textbf{Z}_{{\varvec{\Lambda }}_{\textbf{te}}} = {\varvec{\Lambda }}_{\textbf{t}} \otimes \textbf{Z}\big[{\varvec{\Lambda }}_{\textbf{e}} \otimes \textbf{I}_v\big]$ and **u**_n_ is modelled with a non-separable factor analytic model of order one. The other two models are referred to as the non-separable factor analytic linear mixed model (NFA-LMM) and the unstructured factor analytic linear mixed model (UFA-LMM). The SFA-LMM is used to demonstrate all remaining methods below, but note that similar results can be obtained for the NFA-LMM and UFA-LMM.

Constraints are required in the SFA-LMM to ensure unique solutions for $\textbf{G}$ and **R**. In particular, the top left elements in ${{\varvec{\Lambda }}}_{\textbf{t}}$ (although not strictly required), ${{\varvec{\Psi }}}_{\textbf{t}}$ and **R**_**t**_ are set to one, the upper right triangle in ${{\varvec{\Lambda }}}_{\textbf{t}}$ and/or ${{\varvec{\Lambda }}}_{\textbf{e}}$ are set to zero when $k_t$ and/or $k_e > 1$ and ${\textbf{D}}_{\textbf{t}} \otimes {\textbf{D}}_{\textbf{e}}$ is set to ${\textbf{I}}_{k_{t}} \otimes {\textbf{I}}_{k_{e}}$. Let the loadings and scores with these constraints be denoted by ${{\varvec{\Lambda }}}^*_{\textbf{t}} \otimes {{\varvec{\Lambda }}}^*_{\textbf{e}}$ and ${\textbf{f}}^{\hskip 0.5pt *}_{\textbf{te}}$. The loadings and scores can be rotated back to their original form in Eq. 13 for interpretation:

15 $$\begin{aligned} {{\varvec{\Lambda }}}_{\textbf{t}} = {{\varvec{\Lambda }}}^*_{\textbf{t}} \textbf{V}_{\textbf{t}}\textbf{D}^{-1/2}_{\textbf{t}} \qquad \text {and} \qquad {{\varvec{\Lambda }}}_{\textbf{e}} = {{\varvec{\Lambda }}}^*_{\textbf{e}} \textbf{V}_{\textbf{e}}\textbf{D}^{-1/2}_{\textbf{e}}, \end{aligned}$$

where ${\textbf{f}}_{\textbf{te}} = \big( \textbf{D}^{1/2}_{\textbf{t}}{\textbf{V}}^{\scriptscriptstyle \top }_{\textbf{t}} \otimes \textbf{D}^{1/2}_{\textbf{e}}{\textbf{V}}^{\scriptscriptstyle \top }_{\textbf{e}} \otimes \textbf{I}_v\big) {\textbf{f}}^{\hskip 0.5pt *}_{\textbf{te}}$. The rotation matrices are obtained via the singular value decompositions ${{\varvec{\Lambda }}}^*_{\textbf{t}} = \textbf{U}_{\textbf{t}}\textbf{D}^{1/2}_{\textbf{t}}{\textbf{V}}^{\scriptscriptstyle \top }_{\textbf{t}}$ and ${{\varvec{\Lambda }}}^*_{\textbf{e}} = \textbf{U}_{\textbf{e}}\textbf{D}^{1/2}_{\textbf{e}}{\textbf{V}}^{\scriptscriptstyle \top }_{\textbf{e}}$.

*ASReml-R* obtains REML estimates of the key variance parameters in the SFA-LMM (distinguished by hats) and EBLUPs of the key random effects (distinguished by tildes). A more efficient computational approach for fitting the SFA-LMM is developed in the Supplementary Material. Lastly, note that the phenotypes for each trait were scaled to unit variance to assist convergence in *ASReml-R* and then transformed back to their original scale after estimation.

### Model selection and interpretation

Model selection in the SFA-LMM was achieved using a combination of formal and informal criteria. Formal selection was achieved using the Akaike Information Criterion (AIC) and informal selection was achieved using measures of variance explained by the common factors. The percentage of variance explained for the $h^{th}$ trait by environment combination is given by:

16 $$\begin{aligned} {v}_h = 100 \text { diag}\big ({\hat{{\varvec{\Lambda }}}}_{\textbf{t}}{\hat{\textbf{D}}}_{\textbf{t}}{\hat{{\varvec{\Lambda }}}}_{\textbf{t}}^{\scriptscriptstyle \top } \otimes \hat{{\varvec{\Lambda }}}_{\textbf{e}} {\hat{\textbf{D}}}_{\textbf{e}}{\hat{{\varvec{\Lambda }}}}_{\textbf{e}}^{\scriptscriptstyle \top }\big )_h \oslash \text {diag}\big (\hat{\textbf{G}}_{\textbf{te}}\big )_h, \end{aligned}$$

where $\hat{\textbf{G}}_{\textbf{te}} = \hat{{\varvec{\Lambda }}}_{\textbf{t}} {\hat{\textbf{D}}}_{\textbf{t}}{\hat{{\varvec{\Lambda }}}}_{\textbf{t}}^{\scriptscriptstyle \top }\otimes \hat{{\varvec{\Lambda }}}_{\textbf{e}} {\hat{\textbf{D}}}_{\textbf{e}}{\hat{{\varvec{\Lambda }}}}_{\textbf{e}}^{\scriptscriptstyle \top } + \hat{{\varvec{\Psi }}}_{\textbf{t}}\otimes \hat{{\varvec{\Psi }}}_{\textbf{e}}$ and $\oslash$ is the Hadamard division operator. The overall measure, $\bar{v}$, is then obtained by averaging ${v}_h$ across all *ps* trait by environment combinations. Similar measures are also obtained for the $i^{th}$ trait and $j^{th}$ environment, $v_{t_i}$ and $v_{e_j}$, by averaging ${v}_h$ across all *p* environments and all *s* traits, respectively.

Interpretation from the SFA-LMM was achieved using the overall variance matrices between traits and between environments. The variance matrix between traits for the $j^{th}$ environment is given by:

17 $$\begin{aligned} \bar{\textbf{G}}_{\textbf{t}_j} = \hat{{\varvec{\Lambda }}}_{\textbf{t}} {\hat{\textbf{D}}}_{\textbf{t}}{\hat{{\varvec{\Lambda }}}}_{\textbf{t}}^{\scriptscriptstyle \top }[{\hat{{\varvec{\Lambda }}}_{\textbf{e}_j}} {\hat{\textbf{D}}}_{\textbf{e}}{\hat{{\varvec{\Lambda }}}}^{\scriptscriptstyle \top }_{\textbf{e}_j}] + \hat{{\varvec{\Psi }}}_{\textbf{t}}\hat{\psi }_{{e}_j}, \end{aligned}$$

where $\hat{{\varvec{\Lambda }}}_{\textbf{e}_{j}} = \big [\hat{\lambda }_{e_{j1}} \, \ldots \, \hat{\lambda }_{e_{jk_e}}\big ]$. The variance matrix between environments for the $i^{th}$ trait is given by:

18 $$\begin{aligned} \bar{\textbf{G}}_{\textbf{e}_i} = [{\hat{{\varvec{\Lambda }}}_{\textbf{t}_i}} {\hat{\textbf{D}}}_{\textbf{t}}{\hat{{\varvec{\Lambda }}}}^{\scriptscriptstyle \top }_{\textbf{t}_i}]{\hat{{\varvec{\Lambda }}}}_{\textbf{e}} {\hat{\textbf{D}}}_{\textbf{e}}{\hat{{\varvec{\Lambda }}}}_{\textbf{e}}^{\scriptscriptstyle \top } + \hat{\psi }_{t_i}\hat{{\varvec{\Psi }}}_{\textbf{e}}, \end{aligned}$$

where $\hat{{\varvec{\Lambda }}}_{\textbf{t}_{i}} = \big [\hat{\lambda }_{t_{i1}}\, \ldots \, \hat{\lambda }_{t_{ik_t}}\big ]$. The overall variance matrices are then obtained by averaging $\bar{\textbf{G}}_{\textbf{t}_j}$ across all *p* environments and $\bar{\textbf{G}}_{\textbf{e}_i}$ across all *s* traits.

### Selection tools and index

The selection tools of Smith and Cullis (2018) were extended for the SFA-LMM to enable efficient selection across multiple traits and multiple environments. These tools provide measures of overall performance and stability for each genotype.

The overall performance of all genotypes for the $i^{th}$ trait is given by:

19 $$\begin{aligned} \text {OP}_{i} = \bar{\lambda }_{e_{1}}\tilde{\textbf{f}}_{\textbf{e}_{i1}}, \end{aligned}$$

where $\bar{\lambda }_{e_{1}}$ is the mean estimated loading for the first environmental factor and $\tilde{\textbf{f}}_{\textbf{e}_{i1}}$ is the corresponding *v*-vector of predicted scores, with $\tilde{\textbf{f}}_{\textbf{e}_{i}}= \big (\hat{{\varvec{\Lambda }}}_{\textbf{t}_{i}} \otimes \textbf{I}_{k_e} \otimes \textbf{I}_v\big )\tilde{\textbf{f}}_{\textbf{te}}$. OP$_i$ can therefore be viewed as the expected genotype performance for the $i^{th}$ trait in an average environment. The separate OP$_i$ measures can also be combined across traits to form a selection index given by:

20 $$\begin{aligned} {\mathcal {I}}&= \omega _1\bar{\text {OP}}_1 +\ldots + \omega _s\bar{\text {OP}}_s, \end{aligned}$$

where $\omega _i$ is the weight for the $i^{th}$ trait and $\bar{\text {OP}}_i$ is the corresponding *v*-vector of standardised overall performances, with $\bar{\text {OP}}_i=\tilde{\textbf{f}}_{\textbf{e}_{i1}}\Big /\sqrt{d_{e_1}\hat{{\varvec{\Lambda }}}_{\textbf{t}_{i}}\hat{\textbf{D}}_{\textbf{t}}{\hat{{\varvec{\Lambda }}}}^{\scriptscriptstyle \top }_{\textbf{t}_{i}}}$.

Lastly, the stability of all genotypes for the $i^{th}$ trait is given by:

21 $$\begin{aligned} \text {RMSD}_{i} = \sqrt{\text {diag}\big ({\tilde{\textbf{E}}}_{i}{\tilde{\textbf{E}}}_{i}^{\scriptscriptstyle \top }\big )/p}, \end{aligned}$$

where $\tilde{\textbf{E}}_{i}= \tilde{\textbf{F}}_{\textbf{e}_i}{\hat{{\varvec{\Lambda }}}}^{{\scriptscriptstyle \top }}_{\textbf{e}} - \tilde{\textbf{f}}_{\textbf{e}_{i1}}{\hat{\varvec{\lambda }}}^{{\scriptscriptstyle \top }}_{\textbf{e}_1}$ is the $v\times p$ matrix of additive GET effects corresponding to the higher order factors and $\tilde{\textbf{F}}_{\textbf{e}_i}=\big [\tilde{\textbf{f}}_{\textbf{e}_{i1}} \ \ldots \ \tilde{\textbf{f}}_{\textbf{e}_{i{k_e}}}\big ]$ is the $v\times k_e$ matrix of predicted genotype scores. RMSD$_i$ can therefore be viewed as the variance in genotype performance for the $i^{th}$ trait across all environments. The inclusion of RMSD_*i*_ within the same selection index as OP_*i*_ is the topic of current research.

## Results

This section presents the results from fitting the non-separable, separable and partially separable linear mixed models to the multi-trait MET dataset. The variance models for the additive GET effects are summarised in Table 4. The dataset is summarised in Tables 1 and 2, and comprises 267 genotypes with marker data that were evaluated for three agronomic traits (YLD, DTF, and PHT) across 12 growing environments in the Australian Rice Breeding Program. The results from each model are detailed below, along with the extension of the selection tools for the SFA-LMM.

### Baseline linear mixed models

The analyses began by fitting four baseline linear mixed models which assume the additive GET effects are independent across different trait by environment combinations (Table 5a). These analyses resemble single-trait single-environment analyses that should be performed on multi-trait MET datasets before more complex models are considered. These analyses were used to inspect the experimental design, address spatial variations and identify potential outliers.

The single-trait single-environment analyses were also used to compare the four residual models in Table 3. The non-separable and separable models in *res*$_1$ and *res*$_2$ provide a good fit to the dataset, but note the former does not model the residual correlation between traits and the latter assumes the same residual correlations between columns and rows for all traits in each environment. The separable model in *res*$_3$ provides the poorest fit and is the most restrictive since it assumes the same residual correlation between traits for all environments and the same residual correlations between columns and rows for all traits in each environment. Conversely, the partially separable model in *res*$_4$ provides the best fit and is the least restrictive. This model extends *res*$_3$ to include a specific variance matrix which enables a different residual correlation between traits for each environment and a different residual correlation between columns and rows for each trait by environment combination. The partially separable model in *res*$_4$ was therefore fitted in all subsequent analyses.

### Non-separable factor analytic linear mixed model

A series of NFA-LMMs were then fitted which include a non-separable model for the additive GET effects (Table 5b). These models provide a much better fit than the baseline linear mixed models since they assume the additive GET effects are correlated across different trait by environment combinations. The final order was selected using the formal and informal measures in “Model selection and interpretation”. Three factors $(k = 3)$ were required to achieve an adequate fit and overall percentage of additive genetic variance explained ($\bar{v} = 90.0\%$), with $v_{t_{i}} > 80\%$ and $v_{e_{j}} > 70\%$ for all traits and environments. The 90.0% of variance explained by the common factors reflects GETI common to multiple trait by environment combinations while the remaining 10.0% reflects residual GETI specific to individual traits and environments. Lastly, note that the NFA3 model explained 82.9% of the total genetic variance so that higher order models were not necessary.

### Unstructured factor analytic linear mixed model

A series of UFA-LMMs were then fitted which include a partially separable model for the additive GET effects with fewer variance parameters than the NFA-LMMs (Table 6a). Two environmental factors $(k_{e} = 2)$ were required to achieve an adequate fit and overall percentage of additive genetic variance explained ($\bar{v} = 83.2\%$), with $v_{t_{i}} > 50\%$ and $v_{e_{j}} > 70\%$ for all traits and environments. The 83.2% of variance explained by the common factors reflects GETI common to multiple environments while the remaining 16.8% reflects residual GETI specific to individual traits. Lastly, note that the UFA2 model explained 77.1% of the total genetic variance.

### Partially separable factor analytic linear mixed model

A series of SFA-LMMs were then fitted which include a partially separable model for the additive GET effects with fewer variance parameters than the NFA-LMMs (Table 6b). The SFA-LMM does have more variance parameters than the UFA-LMM for the current dataset, but note that the converse will be true for a more typical number of traits. Three trait factors $(k_t=3)$ and two environmental factors $(k_e=2)$ were required to reach an adequate fit and overall percentage of additive genetic variance explained ($\bar{v} = 83.0\%$), with $v_{t_{i}} > 60\%$ and $v_{e_{j}} > 60\%$ for all traits and environments. The 83.0% of variance explained by the common factors reflects GETI common to multiple traits and multiple environments while the remaining 17.0% reflects residual GETI specific to individual traits and environments. Lastly, note that the SFA3-2 model explained 78.2% of the total genetic variance.

### Model comparison

Formal model selection criteria was used to compare all variance models for the additive GET effects in Table 4. The separable model of Montesinos-López et al. (2016), *mdiag*, is a multi-trait extension of the main effects plus diagonal model. This model provides a poor fit and is restrictive since it assumes the same GEI pattern for each trait and the same GTI pattern for each environment. The separable model of Montesinos-López et al. (2019), *mus*, extends *mdiag* to include an unstructured matrix between traits as well as between environments, but note that this model failed to converge. The partially separable model of Volpato et al. (2019), *comp*, was also fitted, but note that this model provides the poorest fit and is also restrictive since it assumes the same covariance between all environments for each trait. All factor analytic linear mixed models fit the dataset better than *mdiag* and *comp* since they capture different GEI and different GTI patterns, and also fit a more realistic model for GETI. All selected factor analytic linear mixed models were comparable, with the NFA3 model providing the best fit followed by the SFA3-2 model and the UFA2 model. Note, however, the NFA3 model has 3.5–5 times more additive genetic variance parameters than the SFA3-2 and UFA2 models (141 compared to 42 and 31). The SFA3-2 model does have more variance parameters than the UFA2 model for the current dataset since it includes a specific variance for individual traits and environments, rather than just traits (14 compared to 3). These results suggest that the SFA-LMM will provide a superior fit than the UFA-LMM with fewer variance parameters as the number of traits and environments increase. Further interpretation of the SFA3-2 model is presented below.

### Model summaries and interpretation

Table 7 presents a summary of the three agronomic traits and 12 growing environments for the SFA3-2 model. This table shows that the additive genetic variances are different for each trait and environment. The variance for each trait was 0.45 for YLD, 5.32 for DTF and 18.35 for PHT. The variance for each environment was highest for 18MB$_\text {E}$ (16.86) and lowest for 17MB$_\text {L}$ (2.74). By design, the overall variance and the overall variance explained is the same across traits and environments, with 8.04 and 83.0%, respectively. The percentage of variance explained for traits, ${v_{t_i}}$, was 59.0% for YLD, 100.0% for DTF and 90.2% for PHT. The percentage of variance explained for environments, ${v_{e_j}}$, was highest for three Murrumbidgee Valley environments (100.0%) and lowest for 17MV$_\text {L}$ (59.8%) and the three Murray Valley environments in 2018 (62.6–68.4 %). Lastly, Table 7 presents the REML estimates of the constrained factor loadings. These matrices demonstrate the constraints required during estimation (see “Model estimation”).

Table 8 shows that the additive genetic correlations between traits are different for each environment. These correlations were obtained from Eq.  17. The correlations between YLD and DTF were almost zero for all environments, with − 0.02 overall. The correlations between YLD and PHT were highest for 17MV$_{\text {L}}$ (− 0.09) and lowest for 17MV_M_ and three Murrumbidgee Valley environments (− 0.30), with − 0.20 overall. Lastly, the correlations between DTF and PHT were again highest for 17MV$_{\text {L}}$ (− 0.27) and again lowest for 17MV_M_ and the same three Murrumbidgee Valley environments (− 0.33), with − 0.31 overall.

**Fig. 4:**
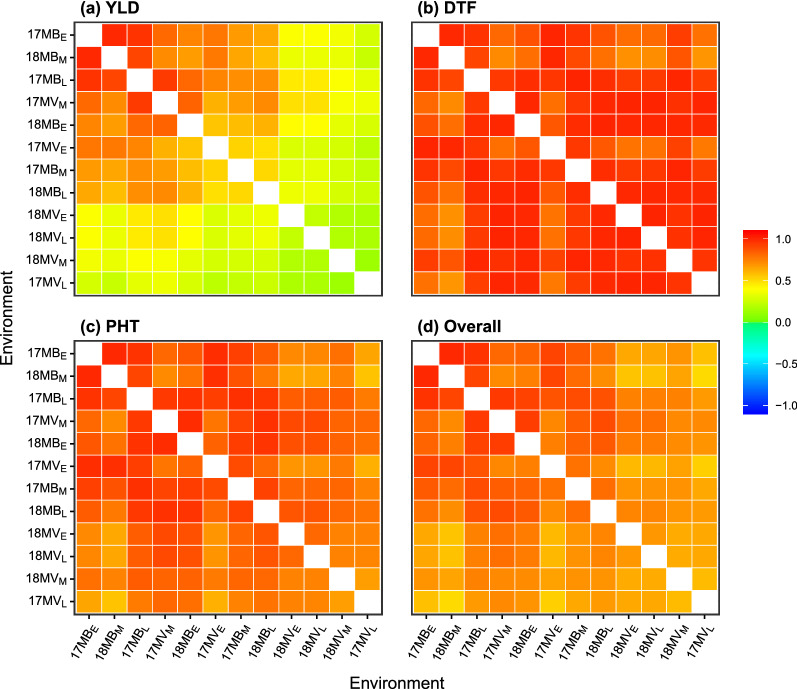
Heatmaps of the additive genetic correlation matrices between environments for **a** grain yield, **b** days to flowering, **c** plant height and **d** overall. All matrices are ordered using the dendrogram applied to **a**. The colourkey ranges from 1 (agreement in rankings) through 0 (dissimilarity in rankings) to − 1 (reversal of rankings)

The heatmaps in Fig. 4 show that the additive genetic correlations between environments are different for each trait. These correlations were obtained from Eq.  18 and are ordered based on a dendrogram applied to YLD (see Cullis et al. 2010). The GEI patterns are substantially different for each trait, especially YLD. The correlations for YLD range from 0.13 to 0.99 (mean of 0.48), for DTF they range from 0.69 to 1.00 (mean of 0.92) and for PHT they range from 0.57 to 0.99 (mean of 0.83). Note that the lowest correlations for YLD correspond to 17MV$_\text {L}$ and the three Murray Valley environments in 2018, which also have the lowest variance explained (see Table 7). The estimated correlations for these environments are therefore an artifact of having low variance explained, and may not reflect the true correlations.

**Fig. 5:**
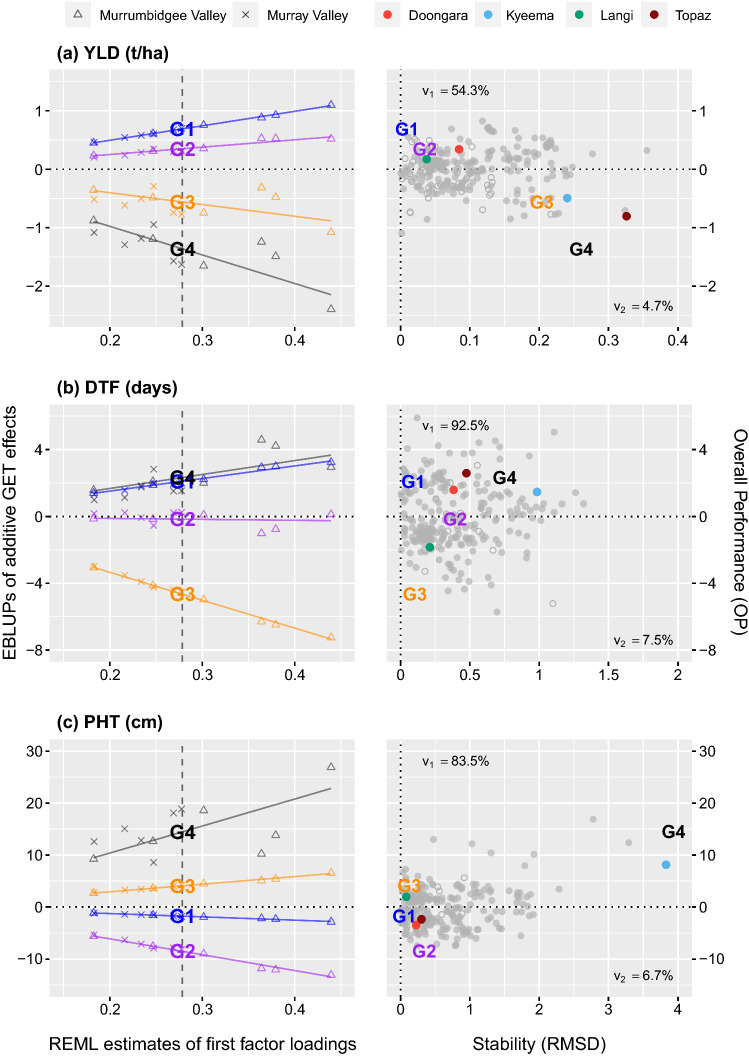
Plant breeding selection tools for **a** grain yield, **b** days to flowering and **c** plant height. The latent regression plots on the left show OP$_i$ for reference genotypes G1-G4, which is given by the fitted value at the mean loading (*vertical dashed line*). The plots on the right show OP$_i$ vs RMSD$_i$ for 267 genotypes, with reference genotypes G1-G4 and four check cultivars distinguished by *colour* and data points distinguished with *closed circles* if the OP$_i$ accuracy is $>0.80$ or *open* otherwise. The variance explained by the first and second factors is also labelled (color figure online)

**Fig. 6:**
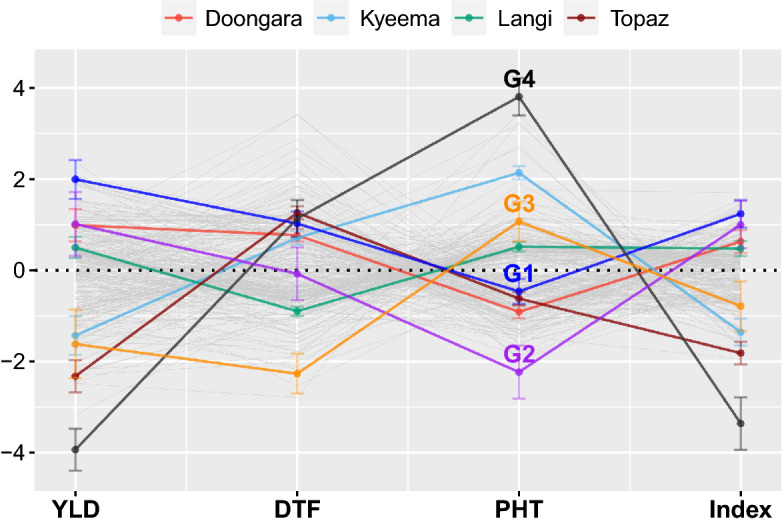
Parallel coordinate plot with standardised $\bar{\text {OP}}_i$ for grain yield, days to flowering and plant height, as well as the selection index with weights 0.7, − 0.2 and − 0.1. The plot includes 267 genotypes, with reference genotypes G1-G4 and four check cultivars distinguished by *colour* and standard errors given by *bars* (color figure online)

Tables 7, 8 and Fig. 4 provide summaries of GETI from the perspective of the traits and environments. The selection tools demonstrated below enable GETI to be summarised from the perspective of the genotypes.

### Selection tools and index

The selection tools were applied to the SFA3-2 model in terms of the additive GET effects. These tools provide breeders with measures of overall performance (OP$_i$) and stability (RMSD$_i$) for each trait. Selection will be demonstrated individually for YLD and then simultaneously for all traits using a selection index.

#### Overall performance and stability

The OP$_i$ measure for YLD is a function of the first factor, which explains 54.3% of the additive genetic variance. Since all loadings for this factor are positive, the fitted GET effects capture non-crossover GEI only (see Smith and Cullis 2018). This feature can be visualised using the latent regression plot in Fig. 5a for reference genotypes G1-G4, where the regression lines diverge and never crossover. The OP$_i$ for each genotype is therefore given by the fitted GET effect at the mean loading of 0.28 (*vertical dashed line*), that is 0.69 for G1, 0.35 for G2, − 0.56 for G3 and − 1.36 t/ha for G4. This measure reflects the expected YLD performance of G1-G4 in an average environment.

The RMSD$_i$ measure for YLD is a function of the second factor, which only explains 4.7% of the additive genetic variance. Since this factor comprises both positive and negative loadings, the regression lines crossover (not shown) and the fitted GET effects predominately capture crossover GEI only. The RMSD$_i$ measure can also be visualised using the latent regression plot in Fig. 5a. The RMSD$_i$ for each genotype is given by the root mean square of the deviations around the first factor regression line, that is 0.01 for G1, 0.03 for G2, 0.20 for G3 and 0.26 t/ha for G4. This measure reflects the variance in YLD performance of G1-G4 across all environments.

Selection across all genotypes for YLD can then be achieved using the OP$_i$ vs RMSD_*i*_ plot in Fig. 5a. Consistently high yielding genotypes occur at the top left of this figure. For example, G1 is high yielding and stable since it has high OP_*i*_ and low RMSD_*i*_. Conversely, low yielding and unstable genotypes which have low OP_*i*_ and high RMSD_*i*_ occur at the bottom right (G4 and Topaz). The remaining highlighted genotypes are above average (G2, Doongara and Langi) or below average (G3 and Kyeema) yielding, with different stabilities. Similar interpretation can be made for DTF and PHT in Fig. 4a and 4b, but note that the first factor explains 92.5 and 83.5 % of the additive genetic variance for these traits. Lastly note that genotypes of high interest occur at the bottom left for these traits, such as G3 which flowers consistently early and G2 which grows consistently short.

#### Selection index

Simultaneous selection across all traits can be achieved using the selection index in Fig. 6. For example, G1 is much higher yielding than G2, however, they have a very similar index since G2 flowers earlier and is much shorter than G1. Both genotypes also have a higher index than the four check cultivars and G3. Conversely, G4 is the lowest yielding genotype and is much taller than all other test genotypes so it has the lowest index. Figure 6 provides breeders with an efficient way to select superior genotypes and examine GTI summarised over environments. Lastly, note that the weights used in this index are arbitrary (0.7 for YLD, − 0.2 for DTF and − 0.1 for PHT) and chosen to illustrate the concepts and methods developed in “Statistical models”.

## Discussion

This paper developed a single-stage GS approach which incorporates information on multiple traits and multiple environments within a partially separable factor analytic framework. The advantage of using both sources of information is that breeders can utilise GETI to obtain more accurate predictions across correlated traits and environments. The partially separable factor analytic linear mixed model (SFA-LMM) developed in this paper was motivated by the need for a more parsimonious alternative to the non-separable factor analytic linear mixed model of Smith et al. (2007) and the unstructured factor analytic linear mixed model of Smith et al. (2019). The utility of the SFA-LMM was compared to GS extensions of these approaches, as well as the two separable approaches of Montesinos-López et al. (2016, 2019) and the partially separable approach of Volpato et al. (2019).

Plant breeders select genotypes with superior performance across a set of production environments for multiple traits of commercial importance. Plant breeding datasets are therefore naturally generated as *multi-trait* MET datasets. Many of the current approaches for GS, however, only include models for GEI in single traits or models for GTI in single environments, but very few have considered appropriate models for GETI across multiple traits and multiple environments. Recently, Montesinos-López et al. (2016) and Montesinos-López et al. (2019) demonstrated separable models for GETI which assume the same GEI pattern for each trait and the same GTI pattern for each environment. Volpato et al. (2019) demonstrated a restrictive partially separable model which assumes the same covariance between environments for each trait. The results of this paper show that the approaches of Montesinos-López et al. (2016) and Volpato et al. (2019) provide a poor fit to the dataset, whereas the approach of Montesinos-López et al. (2019) failed to converge using REML estimation.

The non-separable and partially separable factor analytic linear mixed models include more appropriate models for GETI which enable different GEI and different GET patterns. The NFA-LMM includes a factor analytic matrix that is fitted directly to the individual trait by environment combinations. The UFA-LMM includes the Kronecker product of an unstructured matrix between traits and a factor analytic matrix between environments, with an additional specific variance matrix for traits. In this paper, both approaches were extended for GS to enable direct comparison with the SFA-LMM. The SFA-LMM developed in this paper is an extension of the UFA-LMM to include a factor analytic matrix between traits as well as between environments, with an additional specific variance matrix for traits as well as environments.

The SFA-LMM was compared to the UFA-LMM and NFA-LMM using a multi-trait MET dataset from the Australian Rice Breeding Program. The dataset comprises 267 genotypes that were evaluated in a single late stage for three traits across 12 environments in the south-eastern rice growing region of Australia. This dataset was used to illustrate the concepts and methods developed in “Statistical models”. Note, however, all data relevant to the current genotypes, including previous years and stages, should be considered when constructing a multi-trait MET dataset for selection (Smith et al. 2021). Also note that the current dataset does not include a typical number of traits, which has two important consequences: (i) it does not exploit the dimension reduction feature of the SFA-LMM and (ii) it does not highlight the large number of parameters typical to the UFA-LMM and NFA-LMM. The practical implication of this will now be discussed.

The results of this paper show that all factor analytic linear mixed models provide a good fit to the dataset, with the NFA-LMM providing the best fit followed by the SFA-LMM and the UFA-LMM. These results suggest that the assumption of separability between traits and environments is unlikely to hold true for the current dataset. The NFA-LMM therefore provides the best fit since it does not assume separability and therefore includes the most general framework for GETI. These results match that of Smith et al. (2007). Note, however, the selected NFA-LMM already has 3.5–5 times more additive genetic variance parameters than the selected SFA-LMM and UFA-LMM, and this will increase even further when there is a more typical number of traits. The selected SFA-LMM does have more parameters than the UFA-LMM for the current dataset, but this is because it required all three possible trait factors (which is equivalent to an unstructured matrix) and it includes a specific variance for individual traits and environments (rather than just traits). These results suggest that the SFA-LMM will provide a better fit than the UFA-LMM with fewer variance parameters as the number of traits and environments increase.

There are three important results from fitting the SFA-LMM to the multi-trait MET dataset:

1. The overall percentage of additive genetic variance explained was 83.0%, with 59.0% for YLD, 100.0% for DTF and 90.2% for PHT (Table 7). The percentage of variance explained for environments ranged from 59.8 to 100 %.
2. The additive genetic correlations between traits are higher for 17MV$_\text {L}$ and the three Murray Valley environments in 2018 compared to all other environments (Table 8). The correlations between environments are much higher for DTF and PHT compared to YLD (Fig. 4).
3. The selection index was demonstrated for 267 genotypes in the multi-trait MET dataset.

Each point is now discussed further.

With regards to point 1, the percentage of additive genetic variance explained for YLD was 59.0%, despite fitting three trait factors and two environmental factors. This highlights two important issues. Firstly, Meyer (2009) warned about the challenges of using factor analytic models when the covariances between traits cannot be attributed to a small number of common factors. This is often the case when the correlations between traits are low and/or the number of traits is small. This challenge was observed when applying the SFA-LMM to the current dataset in which all three possible factors were required. Secondly, the 59.0% explained by the common factors most likely represents the percentage of GEI in YLD which is common to DTF and PHT, whereas the remaining 41.0% most likely reflects GEI specific to YLD alone. To a lesser extent, the 59.0 and 41.0 % represent GTI common and specific to individual environments, respectively. In the context of using separable models, these results again highlight the importance of using traits with sufficiently similar GEI patterns (and environments with sufficiently similar GTI patterns). In the context of using the SFA-LMM, these results highlight the importance of including the specific variance matrix which enables different GEI and different GTI patterns and also accounts for any additional lack-of-fit for traits and environments. This approach may become useful when formally assessing the assumption of separability based on the amount of specific variation.

With regards to point 2, understanding the additive genetic correlations between traits and environments provides valuable information for making selection decisions. In terms of traits, the lack of correlation between YLD and DTF (− 0.02) indicates that the current breeding germplasm could be subject to selection for DTF without impacting YLD. For example, there is a need to develop high yielding cultivars for early and late flowering time, and the results suggest that both breeding targets could be pursued with the current germplasm. The negative correlation between YLD and PHT (− 0.20) indicates that shorter genotypes tend to be high yielding. This correlation reflects the breeding objectives where selection has primarily been for high yielding and short genotypes. It could also reflect the interaction between lodging and yield since taller genotypes in the south-eastern growing region are typically more prone to lodging at maturity (Lewin and Heenan 1987). Lastly, the negative correlation between DTF and PHT (− 0.31) indicates that shorter genotypes tend to flower late. This correlation may be an indirect result of the breeding objective, that is selection for high yielding and short genotypes, whereas both early and late flowering genotypes are desired. In terms of environments, the correlations for YLD vary substantially, with no clear year, location or seasonal pattern (Fig. 4). This is consistent with the complex nature of GEI in the south-eastern growing region, which is driven by complex environmental factors such as reproductive cold damage from infrequent cool weather periods during microspore development (Williams and Angus 1994). Conversely, the correlations for DTF and PHT are high, which reflects their generally high line-mean heritability across environments in the breeding program and in rice germplasm more broadly (Wei et al. 2020).

With regards to point 3, rice breeders select genotypes that are consistently high yielding, early flowering and short. These selections can be made for each trait separately using the OP$_i$ vs RMSD$_i$ plots in Fig.  5. For example, genotype G1 would be selected for YLD, G3 would be selected for DTF and G2 would be selected for PHT. This approach is similar to threshold selection, with the exception that genotypes are now selected based on their overall performance as well as their stability (Smith and Cullis 2018). With more than one trait, however, this approach is inefficient and ignores the genetic correlations between traits. The selection index in Fig. 6 weights the importance of individual traits based on the breeding objectives. This index utilises the common information shared across the traits and environments within the SFA-LMM, and thence enables breeders to make efficient selections. In this paper, the selection index was demonstrated using additive OP$_i$ alone since the variance explained by the higher order factors is negligible, especially for YLD (4.7%). This index should be used to advance test genotypes for further evaluation and to select potential parents for future crosses, whereas an index based on total (additive plus non-additive) OP$_i$ should be used to select cultivars for commercial release. The inclusion of RMSD$_i$ within the same selection index is the topic of current research.

The selection index provides important information on the overall merit of test genotypes compared to the check cultivars. The check cultivars provide a baseline for the three traits under selection. For example, Langi is a high yielding and soft cooking long grain cultivar which is broadly adapted across the Australian rice growing area. Doongara is a semi-dwarf *japonica* long grain cultivar that flowers late and can be high yielding under favourable conditions, but is susceptible to reproductive cold damage at all growth stages. Kyeema is a tall jasmine style cultivar that does not have the semi-dwarf trait. Kyeema was superseded by Topaz, which is a semi-dwarf jasmine style cultivar with later flowering time than the other three check cultivars. The selection index highlights numerous test genotypes which have a higher overall merit than the check cultivars (e.g. G1 and G2). This demonstrates the immediate genetic gain that can be made in the Australian rice breeding program. The parallel coordinate plot in Fig. 6 also highlights numerous test genotypes that should be retained in the program as parents to maintain genetic variation in the traits under selection, although they may not be candidates for release.

The SFA-LMM developed in this paper includes a partially separable factor analytic model for GETI, which exploits the appealing features of separable and non-separable models. The utility of the SFA-LMM was compared to non-separable, separable and partially separable models for GS. The results show that the SFA-LMM provides a better fit than the separable approaches and a comparable fit to the non-separable and partially separable approaches. In fact, all factor analytic linear mixed models provide a good fit since they include appropriate models for GETI. The distinguishing feature of the SFA-LMM is that it will include fewer parameters than all other approaches as the number of genotypes, traits and environments increases. This research represents an important continuation in the advancement of statistical analyses of plant breeding datasets, particularly with the advent of high throughput phenotypic data involving a very large number of traits and environments.

### Supplementary Information

Below is the link to the electronic supplementary material.

Supplementary file 1 (pdf 77 KB)

Supplementary file 2 (R 25 KB)

## Data Availability

The data that support the findings of this study and *R* scripts to fit all linear mixed models in Table 3 are available on the New South Wales Government data repository (https://data.nsw.gov.au/data/dataset/mtme-rice-breeding-data). The *R* scripts are also provided in the Supplementary Material.

## Appendix 1: Model separability

This appendix demonstrates the difference between non-separable, separable and partially separable models. The examples below are demonstrated using two factors denoted by *B* and *C*, with $s=2$ and $p=2$ levels. Let *A* denote the factor with levels given by all combinations of *B* and *C* (ordered as *C* within *B*), such that $ps=4$. In this paper, factors *B* and *C* represent traits and environments while factor *A* represents their interaction.

### A.1 Non-separable models

Let $\textbf{A}$ denote the $ps \times ps$ *non-separable* variance matrix for factor *A* given by:

$$\begin{aligned} \textbf{A}= \left[ \begin{array}{cccccc} a_{11} &{} \ a_{12} &{} \ a_{13} &{} \ a_{14} \\ a_{21} &{} \ a_{22} &{} \ a_{23} &{} \ a_{24} \\ a_{31} &{} \ a_{32} &{} \ a_{33} &{} \ a_{34} \\ a_{41} &{} \ a_{42} &{} \ a_{43} &{} \ a_{44} \\ \end{array}\right] , \end{aligned}$$

where $a_{hh}$ is the variance of the $h^{th}$ level of *A* and $a_{hi}=a_{ih}$ is the covariance between the $h^{th}$ and $i^{th}$ level of A. This variance matrix is non-separable since it cannot be represented by the Kronecker product of two matrices. Note that there are $ps(ps+1)/2$ unique variance parameters in $\textbf{A}$.

### A.2 Separable models

Let $\textbf{B}$ and $\textbf{C}$ denote the $s \times s$ and $p \times p$ variance matrices for factors *B* and *C*. The Kronecker product between $\textbf{B}$ and $\textbf{C}$ is a $ps \times ps$ *separable* variance matrix given by:

22 $$\begin{aligned} \textbf{B}\otimes \textbf{C}&= \left[ \begin{array}{cc} b_{11} &{} \ b_{12} \\ b_{21} &{} \ b_{22} \end{array}\right] \otimes \left[ \begin{array}{ccc} c_{11} &{} \ c_{12} \\ c_{21} &{} \ c_{22} \end{array}\right] \nonumber \\&=\left[ \begin{array}{cccc} b_{11}c_{11} &{} \ b_{11}c_{12} &{} \ b_{12}c_{11} &{} \ b_{12}c_{12}\\ b_{11}c_{21} &{} \ b_{11}c_{22} &{} \ b_{12}c_{21} &{} \ b_{12}c_{22}\\ b_{21}c_{11} &{} \ b_{21}c_{12} &{} \ b_{22}c_{11} &{} \ b_{22}c_{12}\\ b_{21}c_{21} &{} \ b_{21}c_{22} &{} \ b_{22}c_{21} &{} \ b_{22}c_{22}\\ \end{array}\right] , \end{aligned}$$

where $b_{ii}c_{jj}$ is the variance of the $i^{th}$ level of *B* and $j^{th}$ level of *C* combination, $b_{hi}c_{jk}=b_{ih}c_{kj}$ is the covariance between the $h^{th}$ level of *B* and $j^{th}$ level of *C* combination and the $i^{th}$ level of *B* and $k^{th}$ level of *C* combination. Note that there are $[s(s+1)+p(p+1)]/2$ unique variance parameters in $\textbf{B}\otimes \textbf{C}$.

### A.3 Partially separable models

Let $\textbf{D}$ denote another $ps\times ps$ variance matrix for factor *A*, which is now assumed to be diagonal. When $\textbf{D}$ is added to the Kronecker product between $\textbf{B}$ and $\textbf{C}$, the result is a $ps \times ps$ *partially separable* variance matrix given by:

$$\begin{aligned}\textbf{B}\otimes \textbf{C} + \textbf{D} &= \left[ \begin{array}{cc} b_{11} &{} \ b_{12} \\ b_{21} &{} \ b_{22} \end{array}\right] \otimes \left[ \begin{array}{ccc} c_{11} &{} \ c_{12} \\ c_{21} &{} \ c_{22} \end{array}\right] + \left[ \begin{array}{cccccc} d_{11} &{} \ 0 &{} \ 0 &{} \ 0 \\ 0 &{} \ d_{22} &{} \ 0 &{} \ 0 \\ 0 &{} \ 0 &{} \ d_{33} &{} \ 0 \\ 0 &{} \ 0 &{} \ 0 &{} \ d_{44} \\ \end{array}\right] \\ &= \left[ \begin{array}{cccc} b_{11}c_{11}+ d_{11} &{} b_{11}c_{12} &{} b_{12}c_{11} &{} b_{12}c_{12}\\ b_{11}c_{21} &{} b_{11}c_{22}+ d_{22} &{} b_{12}c_{21} &{} b_{12}c_{22}\\ b_{21}c_{11} &{} b_{21}c_{12} &{} b_{22}c_{11} + d_{33}&{} b_{22}c_{12}\\ b_{21}c_{21} &{} b_{21}c_{22} &{} b_{22}c_{21} &{} b_{22}c_{22}+ d_{44}\\ \end{array}\right] , \end{aligned}$$

where $b_{ii}c_{jj}$ and $b_{hi}c_{jk}$ are defined in Eq.  22 and $d_{hh}$ is the specific variance for the $h^{th}$ level of *A*. Note that there are $[s(s+1)+p(p+1)+2ps]/2$ unique variance parameters in $\textbf{B}\otimes \textbf{C}+\textbf{D}$. Also note that other (non-diagonal) forms of $\textbf{D}$ can be used where appropriate.

### A.4 Application to the partially separable factor analytic model

The partially separable factor analytic (SFA$k_t$-$k_e$) model is based on a three-way separable structure given by:

23 $$\begin{aligned} \mathbf {G_a}&= \mathbf {G_t} \otimes \mathbf {G_e} \otimes \mathbf {G_g}\nonumber \\&= \big({\varvec{\Lambda }}_{\textbf{t}}\mathbf {D_t}{{\varvec{\Lambda }}}^{\scriptscriptstyle \top }_{\textbf{t}} + {\varvec{\Psi }}_{\textbf{t}}\big) \otimes \big({\varvec{\Lambda }}_{\textbf{e}}\mathbf {D_e}{{\varvec{\Lambda }}}^{\scriptscriptstyle \top }_{\textbf{e}} + {\varvec{\Psi }}_{\textbf{e}}\big) \otimes \mathbf {G_g}, \end{aligned}$$

where $\mathbf {G_t}$ is a $s\times s$ factor analytic matrix between traits with $k_t$ factors, $\mathbf {G_e}$ is a $p\times p$ factor analytic matrix between environments with $k_e$ factors and $\mathbf {G_g}$ is a $v\times v$ genomic relationship matrix between genotypes. Note that the separable structure in Eq.  23 is restrictive because it assumes the same genotype by environment interaction (GEI) pattern for each trait and the same genotype by trait interaction (GTI) pattern for each environment. This can be demonstrated using $s=2$ traits and *p* environments:

24 $$\begin{aligned} \mathbf {G_{a}} = \left[ \begin{array}{cc} \big({\varvec{\Lambda }}_{\textbf{t}_{1}}\mathbf {D_t}{{\varvec{\Lambda }}}^{\scriptscriptstyle \top }_{\textbf{t}_1} + \psi _{t_1}\big) &{} {\varvec{\Lambda }}_{\textbf{t}_1}\mathbf {D_t}{{\varvec{\Lambda }}}^{\scriptscriptstyle \top }_{\textbf{t}_2}\\ {\varvec{\Lambda }}_{\textbf{t}_2}\mathbf {D_t}{{\varvec{\Lambda }}}^{\scriptscriptstyle \top }_{\textbf{t}_1} &{} \big({\varvec{\Lambda }}_{\textbf{t}_{2}}\mathbf {D_t}{{\varvec{\Lambda }}}^{\scriptscriptstyle \top }_{\textbf{t}_2} + \psi _{t_2}\big) \end{array} \right] \otimes \big({\varvec{\Lambda }}_{\textbf{e}}\mathbf {D_e}{{\varvec{\Lambda }}}^{\scriptscriptstyle \top }_{\textbf{e}} + {\varvec{\Psi }}_{\textbf{e}}\big) \otimes \mathbf {G_g}, \end{aligned}$$

where ${{\varvec{\Lambda }}}_{\textbf{t}_{i}} = \big [{\lambda }_{t_{i1}}\, \ldots \, {\lambda }_{t_{ik_t}}\big ]$ is a $k_t$-row-vector of loadings for the $i^{th}$ trait. The GEI pattern for each trait is therefore given by $\big ({\varvec{\Lambda }}_{\textbf{e}}\mathbf {D_e}{{\varvec{\Lambda }}}^{\scriptscriptstyle \top }_{\textbf{e}} + {\varvec{\Psi }}_{\textbf{e}}\big )$ but scaled by $\big ({\varvec{\Lambda }}_{\textbf{t}_{1}}\mathbf {D_t}{{\varvec{\Lambda }}}^{\scriptscriptstyle \top }_{\textbf{t}_1} + \psi _{t_1}\big )$ and $\big ({\varvec{\Lambda }}_{\textbf{t}_{2}}\mathbf {D_t}{{\varvec{\Lambda }}}^{\scriptscriptstyle \top }_{\textbf{t}_2} + \psi _{t_2}\big )$ for traits 1 and 2. The GTI pattern is also the same for each environment.

The SFA$k_t$-$k_e$ model is obtained by modifying Eq.  23 to enable a different GEI pattern for each trait and a different GTI for each environment. This can also be demonstrated using $s=2$ traits and *p* environments:

$$\begin{aligned} \mathbf {G_{a}} = \left( \left[ \begin{array}{cc} {\varvec{\Lambda }}_{\textbf{t}_{1}}\mathbf {D_t}{{\varvec{\Lambda }}}^{\scriptscriptstyle \top }_{\textbf{t}_1} &{} {\varvec{\Lambda }}_{\textbf{t}_1}\mathbf {D_t}{{\varvec{\Lambda }}}^{\scriptscriptstyle \top }_{\textbf{t}_2}\\ {\varvec{\Lambda }}_{\textbf{t}_2}\mathbf {D_t}{{\varvec{\Lambda }}}^{\scriptscriptstyle \top }_{\textbf{t}_1} &{} {\varvec{\Lambda }}_{\textbf{t}_{2}}\mathbf {D_t}{{\varvec{\Lambda }}}^{\scriptscriptstyle \top }_{\textbf{t}_2} \end{array} \right] \otimes {\varvec{\Lambda }}_{\textbf{e}}\mathbf {D_e}{{\varvec{\Lambda }}}^{\scriptscriptstyle \top }_{\textbf{e}} + \left[ \begin{array}{cc} \psi _{t_1} &{} 0 \\ 0 &{} \psi _{t_2} \end{array}\right] \otimes {\varvec{\Psi }}_{\textbf{e}}\right) \otimes \mathbf {G_g}. \end{aligned}$$

The GEI patterns for traits 1 and 2 are now given by $\big [{\varvec{\Lambda }}_{\textbf{t}_{1}}\mathbf {D_t}{{\varvec{\Lambda }}}^{\scriptscriptstyle \top }_{\textbf{t}_1}\big ]{\varvec{\Lambda }}_{\textbf{e}}\mathbf {D_e}{{\varvec{\Lambda }}}^{\scriptscriptstyle \top }_{\textbf{e}} + \psi _{t_1}{\varvec{\Psi }}_{\textbf{e}}$ and $\big [{\varvec{\Lambda }}_{\textbf{t}_{2}}\mathbf {D_t}{{\varvec{\Lambda }}}^{\scriptscriptstyle \top }_{\textbf{t}_2}\big ]{\varvec{\Lambda }}_{\textbf{e}}\mathbf {D_e}{{\varvec{\Lambda }}}^{\scriptscriptstyle \top }_{\textbf{e}} + \psi _{t_2}{\varvec{\Psi }}_{\textbf{e}}$. The GTI pattern is also different for each environment.
