# Supplementary material for: Genomic selection for genotype performance and stability using information on multiple traits and multiple environments
Source: Theor Appl Genet. 2023 Apr 7;136(5):104. doi: 10.1007/s00122-023-04305-1 (PMC10082131; doi:10.1007/s00122-023-04305-1)
Supplement: Supplementary file 1 — (pdf 77 KB) [file 122_2023_4305_MOESM1_ESM.pdf]

Theoretical and Applied Genetics

Supplementary material: Genomic selection for gentoype performance and stability using information on multiple traits and multiple environments

Jon Bancic · Ben Ovenden · Gregor Gorjanc · Daniel J. Tolhurst

Received: 07 Jul 2022/ Accepted: 9 Jan 2023

Key message The inclusion of multiple traits and multiple environments within a partially separable factor analytic approach for genomic selection provides breeders with an informative framework to utilise genotype by environment by trait interaction for efficient selection

D.J. Tolhurst ✉
The Roslin Institute and Royal (Dick) School of Veterinary Studies, University of Edinburgh, Easter Bush, Midlothian United Kingdom
E-mail: D.J.Tolhurst@sms.ed.ac.uk

Sparse implementation of the SFA-LMM

This section extends the sparse implementation of the average information (AI) algorithm for the partially separable factor analytic linear mixed model (SFA-LMM). Thompson et al. (2003) introduced the sparse implementation for factor analytic linear mixed models as a more efficient approach to estimate the key variance parameters, that is the loadings, score variances and specific variances. This approach also provides a natural way to handle cases where some specific variances are zero. When all specific variances are zero, this leads to a (fully) reduced rank factor analytic linear mixed model (Kirkpatrick and Meyer, 2004).

Preliminaries

The SFA-LMM for $\mathbf{y}$, the ns-vector of phenotypic data on v genotypes, p environments and s traits, is:

$$\mathbf{y} = \mathbf{X}\boldsymbol{\tau} + \mathbf{Z}_{\boldsymbol{\Lambda}_{\mathbf{te}}}\mathbf{f}_{\mathbf{te}} + (\mathbf{I}_s \otimes \mathbf{Z})\boldsymbol{\delta}_{\mathbf{te}} + \boldsymbol{\varepsilon}, \qquad (1)$$

where $\mathbf{Z}_{\boldsymbol{\Lambda}_{\mathbf{te}}} = \boldsymbol{\Lambda}_{\mathbf{t}} \otimes \mathbf{Z}[\boldsymbol{\Lambda}_{\mathbf{e}} \otimes \mathbf{I}_v]$ is the $n \times vk_tk_e$ separable factor analytic design matrix, and $\boldsymbol{\varepsilon} = (\mathbf{I}_s \otimes \mathbf{Z})\mathbf{u}_{\mathbf{n}} + (\mathbf{I}_s \otimes \mathbf{Z}_{\mathbf{p}})\mathbf{u}_{\mathbf{p}} + \mathbf{e}$, with $\text{var}(\boldsymbol{\varepsilon}) = \mathbf{R}_{\boldsymbol{\varepsilon}}$. The SFA-LMM in Equation 1 can be extended by reordering and partitioning the regression residuals as $\boldsymbol{\delta}_{\mathbf{te}} = (\boldsymbol{\delta}_{\mathbf{te}_1}^{\top}, \boldsymbol{\delta}_{\mathbf{te}_2}^{\top})^{\top}$, where $\boldsymbol{\delta}_{\mathbf{te}_1}$ is a vp_1s_1-vector with no zero elements and $\boldsymbol{\delta}_{\mathbf{te}_2}$ is a $v(p_1s_2 + p_2s)$-vector with all zero elements, such that $p = p_1 + p_2$ and $s = s_1 + s_2$, with $p_1 \geq 1$ due to the constraints required during estimation (see Section X of the manuscript). Two simpler models can also be obtained:

1. When $p_1 = p$ and $s_1 = s$, no specific variances are zero. This model is the conventional SFA-LMM.
2. When $s_1 = 0$, all specific variances are effectively zero. This model is referred to as the reduced rank SFA-LMM.

The model considered below allows some specific variances to be non-zero and some to be zero. The SFA-LMM can therefore be written as:

$$\mathbf{y} = \mathbf{X}\boldsymbol{\tau} + \mathbf{Z}_{\boldsymbol{\Lambda}_{\mathbf{te}}}\mathbf{f}_{\mathbf{te}} + \mathbf{Z}_1\boldsymbol{\delta}_{\mathbf{te}_1} + \mathbf{Z}_2\boldsymbol{\delta}_{\mathbf{te}_2} + \boldsymbol{\varepsilon}, \qquad (2)$$

where $[\mathbf{Z}_1 \ \mathbf{Z}_2]$ is partitioned conformably with $\boldsymbol{\delta}_{\mathbf{te}}$.

It is assumed that $\boldsymbol{\delta}_{\mathbf{te}_2} = \mathbf{0}$, and that:

$$\begin{bmatrix} \mathbf{f}_{\mathbf{te}} \\ \boldsymbol{\delta}_{\mathbf{te}_1} \end{bmatrix} \sim \text{N}\left(\begin{bmatrix} \mathbf{0} \\ \mathbf{0} \end{bmatrix}, \begin{bmatrix} \mathbf{D}_{\mathbf{t}} \otimes \mathbf{D}_{\mathbf{e}} & \mathbf{0} \\ \mathbf{0} & \boldsymbol{\Psi}_{\mathbf{t}_1} \otimes \boldsymbol{\Psi}_{\mathbf{e}_1} \end{bmatrix} \otimes \mathbf{G}_{\mathbf{g}} \right).$$

In this model, the genotype scores and regression residuals are assumed to be independent. Thompson et al. (2003) also present an equivalent formulation where the random effects are assumed to be dependent. This formulation will be extended for the SFA-LMM in a subsequent paper.

The mixed model equations for the SFA-LMM in Equation 2 are given by:

$$\begin{bmatrix} \mathbf{X}^{\top}\mathbf{R}_{\boldsymbol{\varepsilon}}^{-1}\mathbf{X} & \mathbf{X}^{\top}\mathbf{R}_{\boldsymbol{\varepsilon}}^{-1}\mathbf{Z}_{\boldsymbol{\Lambda}_{\mathbf{te}}} & \mathbf{X}^{\top}\mathbf{R}_{\boldsymbol{\varepsilon}}^{-1}\mathbf{Z}_1 \\ \mathbf{Z}_{\boldsymbol{\Lambda}_{\mathbf{te}}}^{\top}\mathbf{R}_{\boldsymbol{\varepsilon}}^{-1}\mathbf{X} & \mathbf{Z}_{\boldsymbol{\Lambda}_{\mathbf{te}}}^{\top}\mathbf{R}_{\boldsymbol{\varepsilon}}^{-1}\mathbf{Z}_{\boldsymbol{\Lambda}_{\mathbf{te}}} + \mathbf{D}_{\mathbf{t}}^{-1} \otimes \mathbf{D}_{\mathbf{e}}^{-1} \otimes \mathbf{G}_{\mathbf{g}}^{-1} & \mathbf{Z}_{\boldsymbol{\Lambda}_{\mathbf{te}}}^{\top}\mathbf{R}_{\boldsymbol{\varepsilon}}^{-1}\mathbf{Z}_1 \\ \mathbf{Z}_1^{\top}\mathbf{R}_{\boldsymbol{\varepsilon}}^{-1}\mathbf{X} & \mathbf{Z}_1^{\top}\mathbf{R}_{\boldsymbol{\varepsilon}}^{-1}\mathbf{Z}_{\boldsymbol{\Lambda}_{\mathbf{te}}} & \mathbf{Z}_1^{\top}\mathbf{R}_{\boldsymbol{\varepsilon}}^{-1}\mathbf{Z}_1 + \boldsymbol{\Psi}_{\mathbf{t}_1}^{-1} \otimes \boldsymbol{\Psi}_{\mathbf{e}_1}^{-1} \otimes \mathbf{G}_{\mathbf{g}}^{-1} \end{bmatrix} \begin{bmatrix} \hat{\boldsymbol{\tau}} \\ \tilde{\mathbf{f}}_{\mathbf{te}} \\ \tilde{\boldsymbol{\delta}}_{\mathbf{te}_1} \end{bmatrix} = \begin{bmatrix} \mathbf{X}^{\top}\mathbf{R}_{\boldsymbol{\varepsilon}}^{-1}\mathbf{y} \\ \mathbf{Z}_{\boldsymbol{\Lambda}_{\mathbf{te}}}^{\top}\mathbf{R}_{\boldsymbol{\varepsilon}}^{-1}\mathbf{y} \\ \mathbf{Z}_1^{\top}\mathbf{R}_{\boldsymbol{\varepsilon}}^{-1}\mathbf{y} \end{bmatrix}. \qquad (3)$$

Prediction of genotype scores and regression residuals

Following Smith et al. (2019), Equations 2 and 3 can be written as:

$$\mathbf{y} = \mathbf{W}\boldsymbol{\beta} + \boldsymbol{\varepsilon} \quad \text{and} \quad \mathbf{C}\tilde{\boldsymbol{\beta}} = \mathbf{W}^{\top}\mathbf{R}_{\boldsymbol{\varepsilon}}^{-1}\mathbf{y}, \qquad (4)$$

where $\mathbf{W} = [\mathbf{X} \ \mathbf{Z}_{\boldsymbol{\Lambda}_{\mathbf{te}}} \ \mathbf{Z}_1]$ and $\mathbf{C} = \mathbf{W}^{\top}\mathbf{R}_{\boldsymbol{\varepsilon}}^{-1}\mathbf{W} + \mathbf{G}_{\mathbf{c}}^{-1}$, with:

$$\tilde{\boldsymbol{\beta}} = \begin{pmatrix} \hat{\boldsymbol{\tau}} \\ \tilde{\mathbf{f}}_{\mathbf{te}} \\ \tilde{\boldsymbol{\delta}}_{\mathbf{te}_1} \end{pmatrix} \text{ and } \mathbf{G}_{\mathbf{c}}^{-1} = \begin{bmatrix} \mathbf{0} & \mathbf{0} & \mathbf{0} \\ \mathbf{0} & \mathbf{D}_{\mathbf{t}}^{-1} \otimes \mathbf{D}_{\mathbf{e}}^{-1} \otimes \mathbf{G}_{\mathbf{g}}^{-1} & \mathbf{0} \\ \mathbf{0} & \mathbf{0} & \boldsymbol{\Psi}_{\mathbf{t}_1}^{-1} \otimes \boldsymbol{\Psi}_{\mathbf{e}_1}^{-1} \otimes \mathbf{G}_{\mathbf{g}}^{-1} \end{bmatrix}.$$

The BLUPs of the key random effects are obtained via absorption of $\mathbf{C}$ onto $\mathbf{y}^{\top}\mathbf{R}_{\boldsymbol{\varepsilon}}^{-1}\mathbf{y}$, which gives:

$$\tilde{\mathbf{f}}_{\mathbf{te}} = [\mathbf{D}_{\mathbf{t}} \otimes \mathbf{D}_{\mathbf{e}} \otimes \mathbf{G}_{\mathbf{g}}]\mathbf{Z}_{\boldsymbol{\Lambda}_{\mathbf{te}}}^{\top}\mathbf{P}\mathbf{y} \text{ and } \tilde{\boldsymbol{\delta}}_{\mathbf{te}_1} = [\boldsymbol{\Psi}_{\mathbf{t}_1} \otimes \boldsymbol{\Psi}_{\mathbf{e}_1} \otimes \mathbf{G}_{\mathbf{g}}]\mathbf{Z}_1^{\top}\mathbf{P}\mathbf{y},$$

where $\mathbf{P} = \mathbf{R}_{\boldsymbol{\varepsilon}}^{-1} - \mathbf{R}_{\boldsymbol{\varepsilon}}^{-1}\mathbf{W}\mathbf{C}^{-1}\mathbf{W}^{\top}\mathbf{R}_{\boldsymbol{\varepsilon}}^{-1}$ is the $ns \times ns$ residual sum of squares matrix. Absorption of $\mathbf{C}$ also produces the prediction error variance matrices, with:

$$\text{var}(\tilde{\mathbf{f}}_{\mathbf{te}} - \mathbf{f}_{\mathbf{te}}) = \mathbf{C}^{\tilde{\mathbf{f}}_{\mathbf{te}}\tilde{\mathbf{f}}_{\mathbf{te}}} \quad \text{and} \quad \text{var}(\tilde{\boldsymbol{\delta}}_{\mathbf{te}_1} - \boldsymbol{\delta}_{\mathbf{te}_1}) = \mathbf{C}^{\tilde{\boldsymbol{\delta}}_{\mathbf{te}_1}\tilde{\boldsymbol{\delta}}_{\mathbf{te}_1}},$$

where $\mathbf{C}^{\tilde{\mathbf{f}}_{\mathbf{te}}\tilde{\mathbf{f}}_{\mathbf{te}}} = \big[\mathbf{D}_{\mathbf{t}} \otimes \mathbf{D}_{\mathbf{e}} \otimes \mathbf{G}_{\mathbf{g}}\big]\big(\mathbf{I}_{vk_tk_e} - \mathbf{Z}_{\boldsymbol{\Lambda}_{\mathbf{te}}}^{\top}\mathbf{P}\mathbf{Z}_{\boldsymbol{\Lambda}_{\mathbf{te}}}\big[\mathbf{D}_{\mathbf{t}} \otimes \mathbf{D}_{\mathbf{e}} \otimes \mathbf{G}_{\mathbf{g}}\big]\big)$ and $\mathbf{C}^{\tilde{\boldsymbol{\delta}}_{\mathbf{te}_1}\tilde{\boldsymbol{\delta}}_{\mathbf{te}_1}} = \big[\boldsymbol{\Psi}_{\mathbf{t}_1} \otimes \boldsymbol{\Psi}_{\mathbf{e}_1} \otimes \mathbf{G}_{\mathbf{g}}\big]\big(\mathbf{I}_{vps} - \mathbf{Z}_1^{\top}\mathbf{P}\mathbf{Z}_1\big[\boldsymbol{\Psi}_{\mathbf{t}_1} \otimes \boldsymbol{\Psi}_{\mathbf{e}_1} \otimes \mathbf{G}_{\mathbf{g}}\big]\big)$. These matrices are equivalent to the diagonal blocks in $\mathbf{C}^{-1}$ corresponding to $\tilde{\mathbf{f}}_{\mathbf{te}}$ and $\tilde{\boldsymbol{\delta}}_{\mathbf{te}_1}$, respectively.

Lastly, note that the components in the equations above assume the variance parameters are known. The variance parameters are unknown, however, so they must be estimated. The resulting predictions are therefore referred to as EBLUPs.

Estimation of loadings and specific variances

The REML estimates of the key variance parameters are obtained by maximising the residual log-likelihood, which is given by:

$$\begin{aligned} l(\mathbf{y_2}) &= -\tfrac{1}{2}\big(\log|\mathbf{H}| + \log|\mathbf{X}^{\top}\mathbf{H}^{-1}\mathbf{X}| + \mathbf{y}^{\top}\mathbf{P}\mathbf{y}\big) \\ &= -\tfrac{1}{2}\big(\log|\mathbf{H}| + \log|\mathbf{X}^{\top}\mathbf{H}^{-1}\mathbf{X}| + \mathbf{y}_{\mathbf{r}}^{\top}\mathbf{H}^{-1}\mathbf{y}_{\mathbf{r}}\big), \end{aligned} \qquad (5)$$

where $\mathbf{y}_2 = \mathbf{L}_2^{\top}\mathbf{y}$ such that $\mathbf{L}_2^{\top}\mathbf{X} = \mathbf{0}$ and $\mathbf{y}_{\mathbf{r}} = \mathbf{y} - \mathbf{X}\hat{\boldsymbol{\tau}}$, with $\text{var}(\mathbf{y}_{\mathbf{r}}) = \mathbf{H}$ (Verbyla, 1990). In particular, the REML estimates are obtained by solving a set of (score) equations, which are given by:

$$s(\boldsymbol{\kappa}) = \frac{\partial l(\mathbf{y}_2)}{\partial \boldsymbol{\kappa}^{\top}} = \mathbf{0}, \qquad (6)$$

where $\boldsymbol{\kappa}$ is the b-vector of variance parameters in the IFA-LMM. The score equations were originally solved using numerical approaches based on the observed or

expected information. The AI algorithm is based on the average information, and computes updates as:

$$\boldsymbol{\kappa}^{(m+1)} = \boldsymbol{\kappa}^{(m)} + \left[\boldsymbol{\mathcal{I}}_{\mathbf{a}}^{(m)}\right]^{-1} \mathbf{s}(\boldsymbol{\kappa}^{(m)}), \tag{7}$$

where $\boldsymbol{\kappa}^{(m)}$ is the b-vector of variance parameters, $\boldsymbol{\mathcal{I}}_{\mathbf{a}}^{(m)}$ is the AI matrix and $\mathbf{s}(\boldsymbol{\kappa}^{(m)})$ is the score equations for the mth iteration. Note that the superscript "m" is removed in the following for brevity. The score equation for κ_i is given by (Gilmour et al., 1995):

$$s(\kappa_i) = -\tfrac{1}{2}\left[\mathrm{tr}\big(\mathbf{P}\dot{\mathbf{H}}_i\big) - \mathbf{y}^\top \mathbf{P}\mathbf{q}_i\right], \tag{8}$$

where $\dot{\mathbf{H}}_i = \frac{\partial \mathbf{H}}{\partial \kappa_i}$ and $\mathbf{q}_i$ is the working variate for the ith variance parameter, which is given by:

$$\mathbf{q}_i = \dot{\mathbf{H}}_i\mathbf{P}\mathbf{y}. \tag{9}$$

Next, let the vectors of key variance parameters in the SFA-LMM be denoted by:

$$\boldsymbol{\lambda}_{\mathbf{a}} = \mathrm{vect}(\boldsymbol{\Lambda}_{\mathbf{a}}),\ \ \mathbf{d}_{\mathbf{a}} = \mathrm{diag}(\mathbf{D}_{\mathbf{a}}) \text{ and } \boldsymbol{\psi}_{\mathbf{a}_1} = \mathrm{diag}(\boldsymbol{\Psi}_{\mathbf{a}_1}),$$

where $a = t$ and e for traits and environments.

The working variates for the key variance parameters are given by:

$$\mathbf{q}_i = \begin{cases} \left[\big(\boldsymbol{\Lambda}_{\mathbf{t}}\mathbf{D}_{\mathbf{t}}\dot{\boldsymbol{\Lambda}}_{\mathbf{t}_i}^\top + \dot{\boldsymbol{\Lambda}}_{\mathbf{t}_i}\mathbf{D}_{\mathbf{t}}\boldsymbol{\Lambda}_{\mathbf{t}}^\top\big) \otimes \mathbf{Z}\big(\boldsymbol{\Lambda}_{\mathbf{e}}\mathbf{D}_{\mathbf{e}}\boldsymbol{\Lambda}_{\mathbf{e}}^\top \otimes \mathbf{G}_{\mathbf{g}}\big)\mathbf{Z}^\top\right]\mathbf{P}\mathbf{y} & \kappa_i \in \boldsymbol{\lambda}_{\mathbf{t}} \\ \left[\boldsymbol{\Lambda}_{\mathbf{t}}\mathbf{D}_{\mathbf{t}}\boldsymbol{\Lambda}_{\mathbf{t}}^\top \otimes \mathbf{Z}\big([\boldsymbol{\Lambda}_{\mathbf{e}}\mathbf{D}_{\mathbf{e}}\dot{\boldsymbol{\Lambda}}_{\mathbf{e}_i}^\top + \dot{\boldsymbol{\Lambda}}_{\mathbf{e}_i}\mathbf{D}_{\mathbf{e}}\boldsymbol{\Lambda}_{\mathbf{e}}^\top] \otimes \mathbf{G}_{\mathbf{g}}\big)\mathbf{Z}^\top\right]\mathbf{P}\mathbf{y} & \kappa_i \in \boldsymbol{\lambda}_{\mathbf{e}} \\ \mathbf{Z}_{\boldsymbol{\Lambda}_{\mathbf{te}}}\left[\dot{\mathbf{D}}_{\mathbf{t}_i} \otimes \mathbf{D}_{\mathbf{e}} \otimes \mathbf{G}_{\mathbf{g}}\right]\mathbf{Z}_{\boldsymbol{\Lambda}_{\mathbf{te}}}^\top\mathbf{P}\mathbf{y} & \kappa_i \in \mathbf{d}_{\mathbf{t}} \\ \mathbf{Z}_{\boldsymbol{\Lambda}_{\mathbf{te}}}\left[\mathbf{D}_{\mathbf{t}} \otimes \dot{\mathbf{D}}_{\mathbf{e}_i} \otimes \mathbf{G}_{\mathbf{g}}\right]\mathbf{Z}_{\boldsymbol{\Lambda}_{\mathbf{te}}}^\top\mathbf{P}\mathbf{y} & \kappa_i \in \mathbf{d}_{\mathbf{e}} \\ \mathbf{Z}_1\left[\dot{\boldsymbol{\Psi}}_{\mathbf{t}_{1_i}} \otimes \boldsymbol{\Psi}_{\mathbf{e}_1} \otimes \mathbf{G}_{\mathbf{g}}\right]\mathbf{Z}_1^\top\mathbf{P}\mathbf{y} & \kappa_i \in \boldsymbol{\psi}_{\mathbf{t}_1} \\ \mathbf{Z}_1\left[\boldsymbol{\Psi}_{\mathbf{t}_1} \otimes \dot{\boldsymbol{\Psi}}_{\mathbf{e}_{1_i}} \otimes \mathbf{G}_{\mathbf{g}}\right]\mathbf{Z}_1^\top\mathbf{P}\mathbf{y} & \kappa_i \in \boldsymbol{\psi}_{\mathbf{e}_1} \end{cases}$$

where $\dot{\boldsymbol{\Lambda}}_{\mathbf{a}_i} = \frac{\partial \boldsymbol{\Lambda}_{\mathbf{a}}}{\partial \lambda_{a_i}}$, $\dot{\mathbf{D}}_{\mathbf{a}_i} = \frac{\partial \mathbf{D}_{\mathbf{a}}}{\partial d_{a_i}}$ and $\dot{\boldsymbol{\Psi}}_{\mathbf{a}_{1_i}} = \frac{\partial \boldsymbol{\Psi}_{\mathbf{a}_1}}{\partial \psi_{a_{1_i}}}$, with $a = t$ or e. The working variates for the specific variances can be simplified since the regression residuals are independent across trait by environment combinations. It therefore follows that:

$$\mathbf{q}_i = \begin{cases} \mathbf{Z}_{1.i}\left[\boldsymbol{\Psi}_{\mathbf{e}_1} \otimes \mathbf{G}_{\mathbf{g}}\right]\mathbf{Z}_{1_i}^\top\mathbf{P}_{i\cdot}\mathbf{y} & \kappa_i \in \boldsymbol{\psi}_{\mathbf{t}_1} \\ \mathbf{Z}_{1.i}\left[\boldsymbol{\Psi}_{\mathbf{t}_1} \otimes \mathbf{G}_{\mathbf{g}}\right]\mathbf{Z}_{1_i}^\top\mathbf{P}_{i\cdot}\mathbf{y} & \kappa_i \in \boldsymbol{\psi}_{\mathbf{e}_1} \end{cases}$$

where $\mathbf{Z}_{1_i}$ is the $n_1 \times vp_1$ design matrix for the ith trait with $n_1 = \sum_{i=1}^{p_1} n_i$ or $\mathbf{Z}_{1_i}$ is the $n_i s_1 \times vs_1$ design matrix for the ith environment, $\mathbf{P}_{i\cdot}$ are the corresponding n_1 or $n_i s_1$ rows in $\mathbf{P}$ and $\mathbf{Z}_{1\cdot i}$ are the corresponding vp_1 or vs_1 columns in $\mathbf{Z}_1$.

The trace term in Equation 8 is then given by:

$$\mathrm{tr}(\mathbf{P}\dot{\mathbf{H}}_i) = \begin{cases} 2\ \mathrm{tr}\left[\big(\dot{\boldsymbol{\Lambda}}_{\mathbf{t}_i}^\top \otimes [\boldsymbol{\Lambda}_{\mathbf{e}}^\top \otimes \mathbf{I}_v]\,\mathbf{Z}^\top\big)\mathbf{R}_{\boldsymbol{\varepsilon}}^{-1}\mathbf{W}\mathbf{C}^{\cdot\tilde{\mathbf{f}}_{\mathbf{te}}}\right] & \kappa_i \in \boldsymbol{\lambda}_{\mathbf{t}} \\ 2\mathrm{tr}\left[\big(\boldsymbol{\Lambda}_{\mathbf{t}}^\top \otimes [\dot{\boldsymbol{\Lambda}}_{\mathbf{e}_i}^\top \otimes \mathbf{I}_v]\mathbf{Z}^\top\big)\mathbf{R}_{\boldsymbol{\varepsilon}}^{-1}\mathbf{W}\mathbf{C}^{\cdot\tilde{\mathbf{f}}_{\mathbf{te}}}\right] & \kappa_i \in \boldsymbol{\lambda}_{\mathbf{e}} \\ \mathrm{tr}\left[\big(\dot{\mathbf{D}}_{\mathbf{t}_i}\mathbf{D}_{\mathbf{t}}^{-1} \otimes \mathbf{D}_{\mathbf{e}} \otimes \mathbf{I}_v\big)\mathbf{Z}_{\boldsymbol{\Lambda}_{\mathbf{te}}}^\top\mathbf{R}_{\boldsymbol{\varepsilon}}^{-1}\mathbf{W}\mathbf{C}^{\cdot\tilde{\mathbf{f}}_{\mathbf{te}}}\right] & \kappa_i \in \mathbf{d}_{\mathbf{t}} \\ \mathrm{tr}\left[\big(\mathbf{D}_{\mathbf{t}} \otimes \dot{\mathbf{D}}_{\mathbf{e}_i}\mathbf{D}_{\mathbf{e}}^{-1} \otimes \mathbf{I}_v\big)\mathbf{Z}_{\boldsymbol{\Lambda}_{\mathbf{te}}}^\top\mathbf{R}_{\boldsymbol{\varepsilon}}^{-1}\mathbf{W}\mathbf{C}^{\cdot\tilde{\mathbf{f}}_{\mathbf{te}}}\right] & \kappa_i \in \mathbf{d}_{\mathbf{e}} \\ \mathrm{tr}\left[\big(\dot{\boldsymbol{\Psi}}_{\mathbf{t}_{1_i}}\boldsymbol{\Psi}_{\mathbf{t}_1}^{-1} \otimes \boldsymbol{\Psi}_{\mathbf{e}_1} \otimes \mathbf{I}_v\big)\mathbf{Z}_1^\top\mathbf{R}_{\boldsymbol{\varepsilon}}^{-1}\mathbf{W}\mathbf{C}^{\cdot\tilde{\boldsymbol{\delta}}_{\mathbf{te}_1}}\right] & \kappa_i \in \boldsymbol{\psi}_{\mathbf{t}_1} \\ \mathrm{tr}\left[\big(\boldsymbol{\Psi}_{\mathbf{t}_1} \otimes \dot{\boldsymbol{\Psi}}_{\mathbf{e}_i}\boldsymbol{\Psi}_{\mathbf{e}}^{-1} \otimes \mathbf{I}_v\big)\mathbf{Z}_1^\top\mathbf{R}_{\boldsymbol{\varepsilon}}^{-1}\mathbf{W}\mathbf{C}^{\cdot\tilde{\boldsymbol{\delta}}_{\mathbf{te}_1}}\right] & \kappa_i \in \boldsymbol{\psi}_{\mathbf{e}_1} \end{cases}$$

where $\mathbf{C}^{\cdot\tilde{\mathbf{f}}_{\mathbf{te}}}$ is the column in the inverse coefficient matrix corresponding to $\tilde{\mathbf{f}}_{\mathbf{te}}$ and $\mathbf{C}^{\cdot\tilde{\boldsymbol{\delta}}_{\mathbf{te}_1}}$ is the column corresponding to $\tilde{\boldsymbol{\delta}}_{\mathbf{te}_1}$.

The trace terms for the specific variances can be further simplified as:

$$\mathrm{tr}(\mathbf{P}\dot{\mathbf{H}}_i) = \begin{cases} \frac{1}{\psi_{t_{1_i}}}\ \mathrm{tr}\left[\big(\boldsymbol{\Psi}_{\mathbf{e}_1} \otimes \mathbf{I}_v\big)\mathbf{Z}_{1_i}^\top\mathbf{R}_{\boldsymbol{\varepsilon}_i}^{-1}\mathbf{W}_{i\cdot}\mathbf{C}^{\cdot\tilde{\boldsymbol{\delta}}_{\mathbf{te}_{1_i}}}\right] & \kappa_i \in \boldsymbol{\psi}_{\mathbf{t}_1} \\ \frac{1}{\psi_{e_{1_i}}}\ \mathrm{tr}\left[\big(\boldsymbol{\Psi}_{\mathbf{t}_1} \otimes \mathbf{I}_v\big)\mathbf{Z}_{1_i}^\top\mathbf{R}_{\boldsymbol{\varepsilon}_i}^{-1}\mathbf{W}_{i\cdot}\mathbf{C}^{\cdot\tilde{\boldsymbol{\delta}}_{\mathbf{te}_{1_i}}}\right] & \kappa_i \in \boldsymbol{\psi}_{\mathbf{e}_1} \end{cases}$$

where $\mathbf{R}_{\boldsymbol{\varepsilon}_i}$ is the ith diagonal block in $\mathbf{R}_{\boldsymbol{\varepsilon}}$, $\mathbf{W}_{i\cdot}$ are the n_1 or $n_i s_1$ rows in $\mathbf{W}$ corresponding to the ith trait or environment and $\mathbf{C}^{\cdot\tilde{\boldsymbol{\delta}}_{\mathbf{te}_{1_i}}}$ are the vp_1 or vs_1 columns in $\mathbf{C}$ corresponding to $\tilde{\boldsymbol{\delta}}_{\mathbf{te}_{1_i}}$. The trace terms avoid working with the dense genomic relationship matrix. When $\mathbf{G}_{\mathbf{g}}$ is not prohibitively large, the trace term for the specific variances can also be computed as:

$$\mathrm{tr}(\mathbf{P}\dot{\mathbf{H}}_i) = \begin{cases} \frac{v}{\psi_{t_{1_i}}}\ \mathrm{tr}\big(\boldsymbol{\Psi}_{\mathbf{e}_1}\big) - \mathrm{tr}\left[\big(\boldsymbol{\Psi}_{\mathbf{e}_1} \otimes \mathbf{G}_{\mathbf{g}}\big)\mathbf{C}^{\boldsymbol{\eta}_{1_i}\boldsymbol{\eta}_{1_i}}\right] & \kappa_i \in \boldsymbol{\psi}_{\mathbf{t}_1} \\ \frac{v}{\psi_{e_{1_i}}}\ \mathrm{tr}\big(\boldsymbol{\Psi}_{\mathbf{t}_1}\big) - \mathrm{tr}\left[\big(\boldsymbol{\Psi}_{\mathbf{t}_1} \otimes \mathbf{G}_{\mathbf{g}}\big)\mathbf{C}^{\boldsymbol{\eta}_{1_i}\boldsymbol{\eta}_{1_i}}\right] & \kappa_i \in \boldsymbol{\psi}_{\mathbf{e}_1} \end{cases}$$

where $\mathbf{C}^{\boldsymbol{\eta}_{1_i}\boldsymbol{\eta}_{1_i}}$ is the prediction error variance matrix of $\boldsymbol{\eta}_{1_i} = \mathbf{Z}_{1_i}^\top\mathbf{P}_{i\cdot}\mathbf{y}$.

Lastly, the AI matrix in Equation 7 is given by:

$$\boldsymbol{\mathcal{I}}_{\mathbf{a}} = \tfrac{1}{2}\mathbf{Q}^\top\mathbf{P}\mathbf{Q}, \tag{10}$$

where $\mathbf{Q} = [\mathbf{q}_1\ \mathbf{q}_2\ \ldots\ \mathbf{q}_b]$ is the $ns \times b$ matrix of working variates across all b variance parameters. The AI matrix is obtained via absorption of $\mathbf{C}$ onto $\mathbf{Q}^\top\mathbf{R}_{\boldsymbol{\varepsilon}}^{-1}\mathbf{Q}$ (Smith, 1999).

References

Gilmour AR, Thompson R, Cullis BR (1995) Average information reml: An efficient algorithm for variance parameter estimation in linear mixed models. Biometrics 51:1440–1450, URL http://doi.org/10.2307/2533274

Kirkpatrick M, Meyer K (2004) Direct estimation of genetic principal components: Simplified analysis of complex phenotypes. Genetics 168:2295–2306, URL http://doi.org/10.1534/genetics.104.029181

Smith AB (1999) Multiplicative mixed models for the analysis of multi-environment trial data. PhD thesis, University of Adelaide, URL http://hdl.handle.net/2440/19539

Smith AB, Borg LM, Gogel BJ, Cullis BR (2019) Estimation of factor analytic mixed models for the analysis of multi-treatment multi-environment trial data. International Biometric Society 24:573–588, URL http://doi.org/10.1007/s13253-019-00362-6

Thompson R, Cullis BR, Smith AB, Gilmour AR (2003) A sparse implementation of the average information algorithm for factor analytic and reduced rank variance models. Australian and New Zealand Journal of Statistics 45:445–459, URL http://doi.org/10.1111/1467-842X.00297

Verbyla AP (1990) A Conditional Derivation of Residual Maximum Likelihood. Australian Journal of Statistics 32:227–230, URL http://dx.doi.org/10.1111/j.1467-842X.1990.tb01015.x
